# Supplementary material for: Localized Translation of gurken/TGF-α mRNA during Axis Specification Is Controlled by Access to Orb/CPEB on Processing Bodies
Source: Cell Rep. 2016 Mar 3;14(10):2451–62. doi: 10.1016/j.celrep.2016.02.038 (PMC4823467; doi:10.1016/j.celrep.2016.02.038)
Supplement: Document S1. Figures S1–S5 [file mmc1.pdf]

Cell Reports, Volume *14*

Supplemental Information

Localized Translation of *gurken/TGF-α* mRNA during Axis Specification Is Controlled by Access to Orb/CPEB on Processing Bodies

Alexander Davidson, Richard M. Parton, Catherine Rabouille, Timothy T. Weil, and Ilan Davis

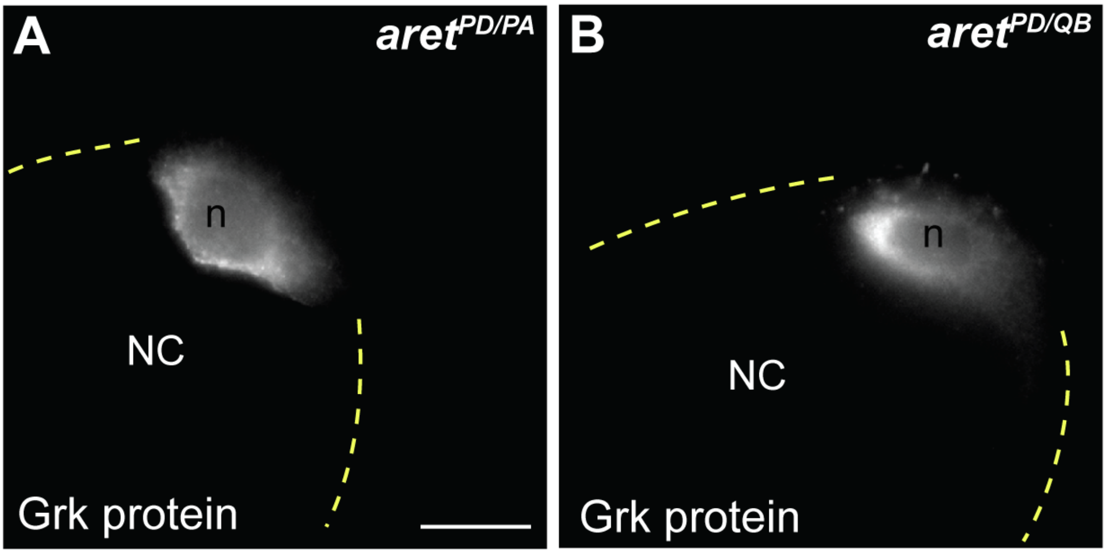

Figure S1. Grk protein is not expressed in the nurse cells in a number of *aret* allelic combinations, related to Figure 1

(A-B) In *aret* mutants, *WT* Grk expression is observed, with no ectopic staining in the nurse cells (A, n = 60; B, n = 60). Scale bar 15 µm; NC, Nurse cells; n, oocyte nucleus. Dashed yellow lines indicate the edges of the egg chamber.

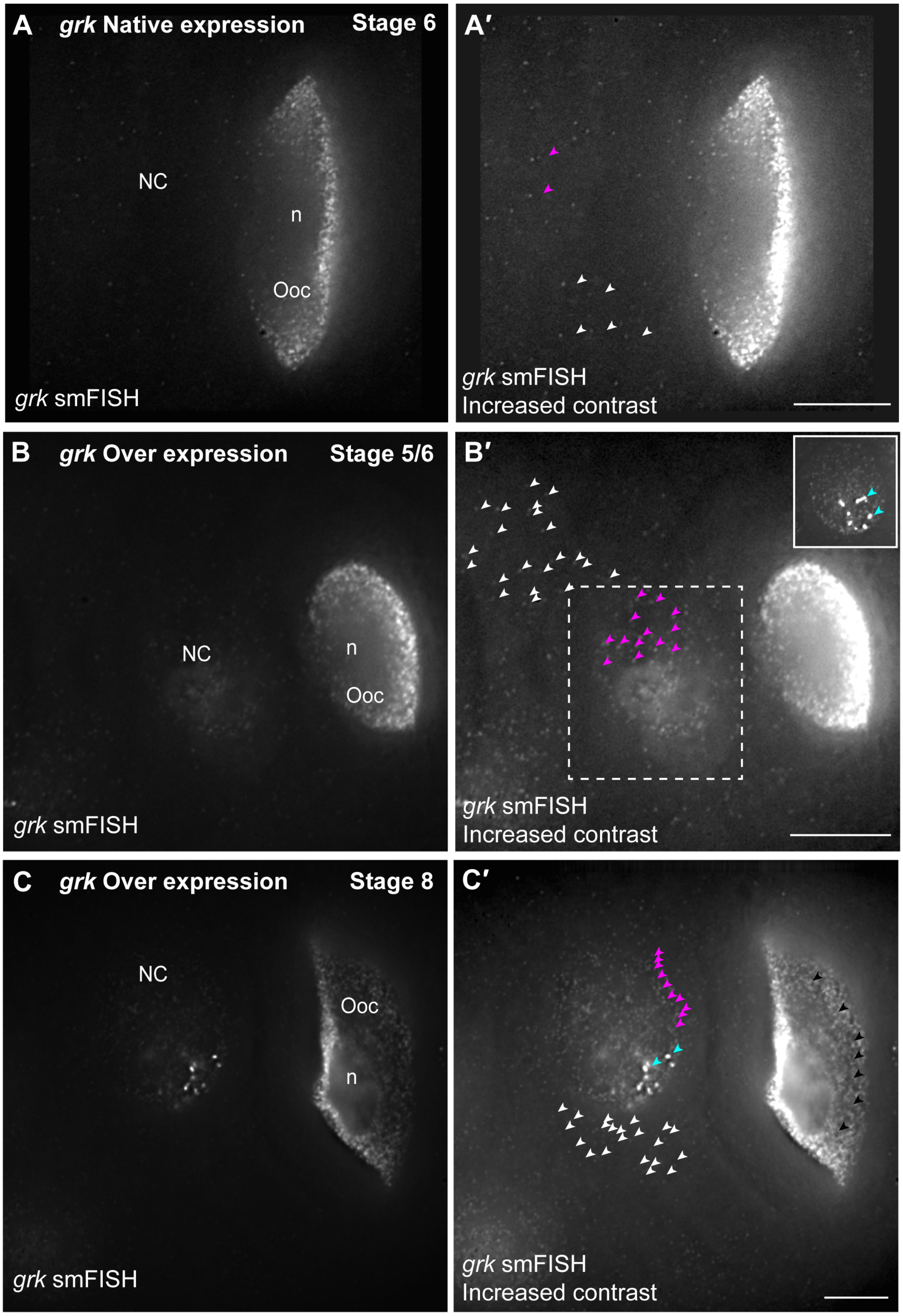

Figure S2. High levels of full-length *grk* transcripts are produced in nurse cells and egg chambers using the UAS-Gal4 system, related to Figure 1

(A-A′) *grk* sm-FISH on *WT* egg chambers showing low numbers of transcripts in NC compared to the oocyte, (A′) is over contrasted to show the weak signal in NC. Arrowheads identify different populations of transcripts in the NC cytoplasm (white) and NC nucleus (magenta). (B-C′)

Overexpression of *grk* driven by the UAS-Gal4 system in nurse cells and oocytes at both early (B-B′) and mid oogenesis (C-C′). Inset in (B′) a different focal plane, corresponding to the dashed box in the main panel, highlighting abundant nascent transcripts in the NC nucleus with over-expression. The increase in numbers of transcript foci between *WT* and driven UASp-*grk* was quantified for regions of interest in the cytoplasm of NC from stage 8 egg chambers: *WT* mean count = 11.28 ±1.3 SEM; driven UASp-*grk* mean count = 29.14 ±3.3 SEM, n = 14, P = 0.00009 (Student's t-test). Arrowheads identify different populations of transcripts in the NC cytoplasm (white), NC nucleus (magenta), nascent transcripts (cyan) and transcripts in the oocyte (black). Scale bars 15 µm; NC, Nurse cells; Ooc, Oocyte; n, oocyte nucleus.

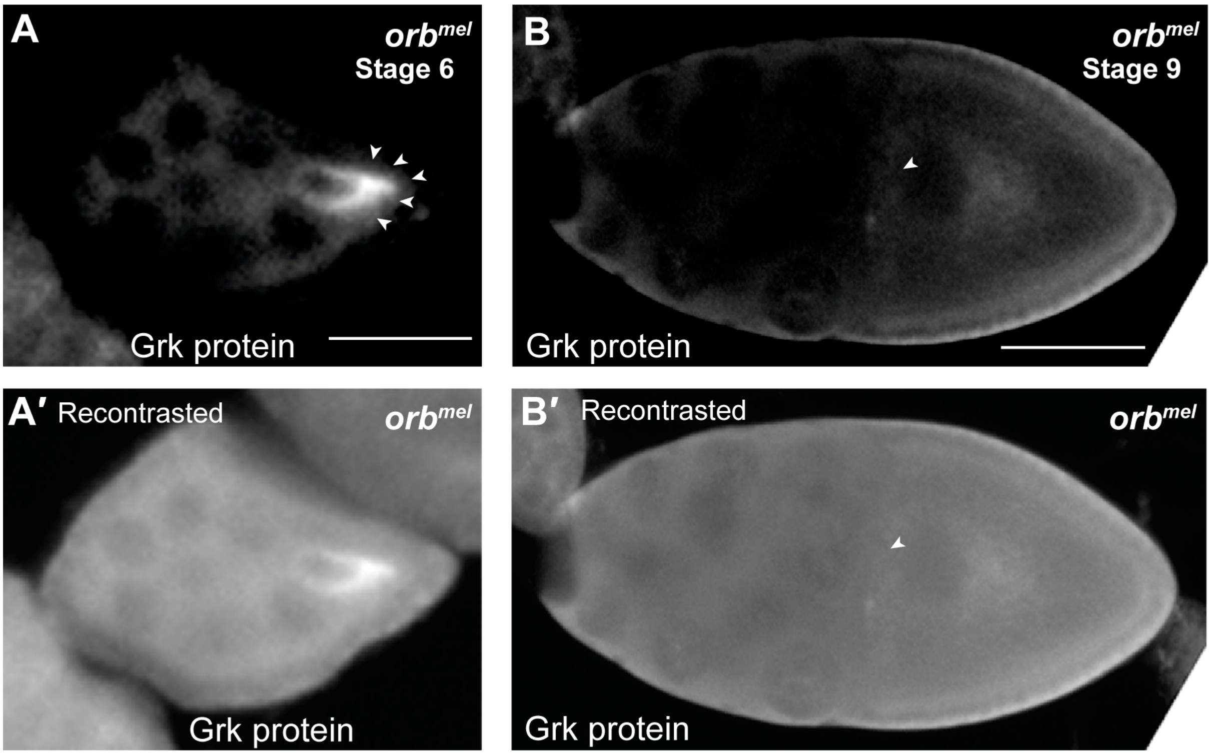

Figure S3. Grk protein is not expressed in late stage *orb^mel* egg chambers, related to Figure 1

(A-B′) Grk antibody staining in orb^{mel} egg chambers. (A-A′) In stage 6 orb^{mel} egg chambers, Grk protein is expressed in the oocyte (n = 5). (B-B′) In stage 9 orb^{mel} egg chambers, Grk protein is not expressed in the oocyte, arrowhead in (B) indicates the DA where expression would be expected (n = 5). (A′ and B′) Over-contrasted images highlighting the lack of Grk. Scale bars 25 µm (A), 50 µm (B).

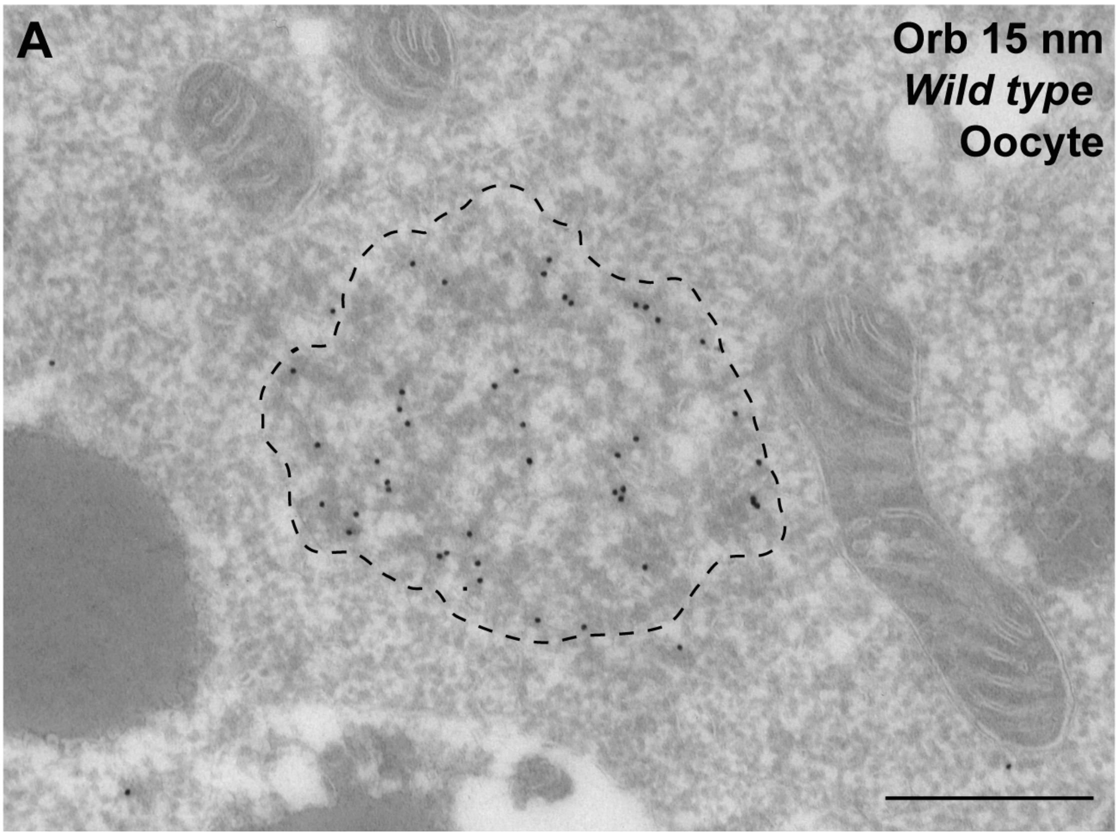

Figure S4. P bodies throughout the oocyte contain Orb protein, related to Figure 3

(A) Orb protein detection by IEM on ultra-thin frozen sections of nurse cells. Anti-Orb (15nm gold) is present in P bodies throughout the oocyte. Dashed black lines mark the edge of the P body. Scale bar: 500nm.

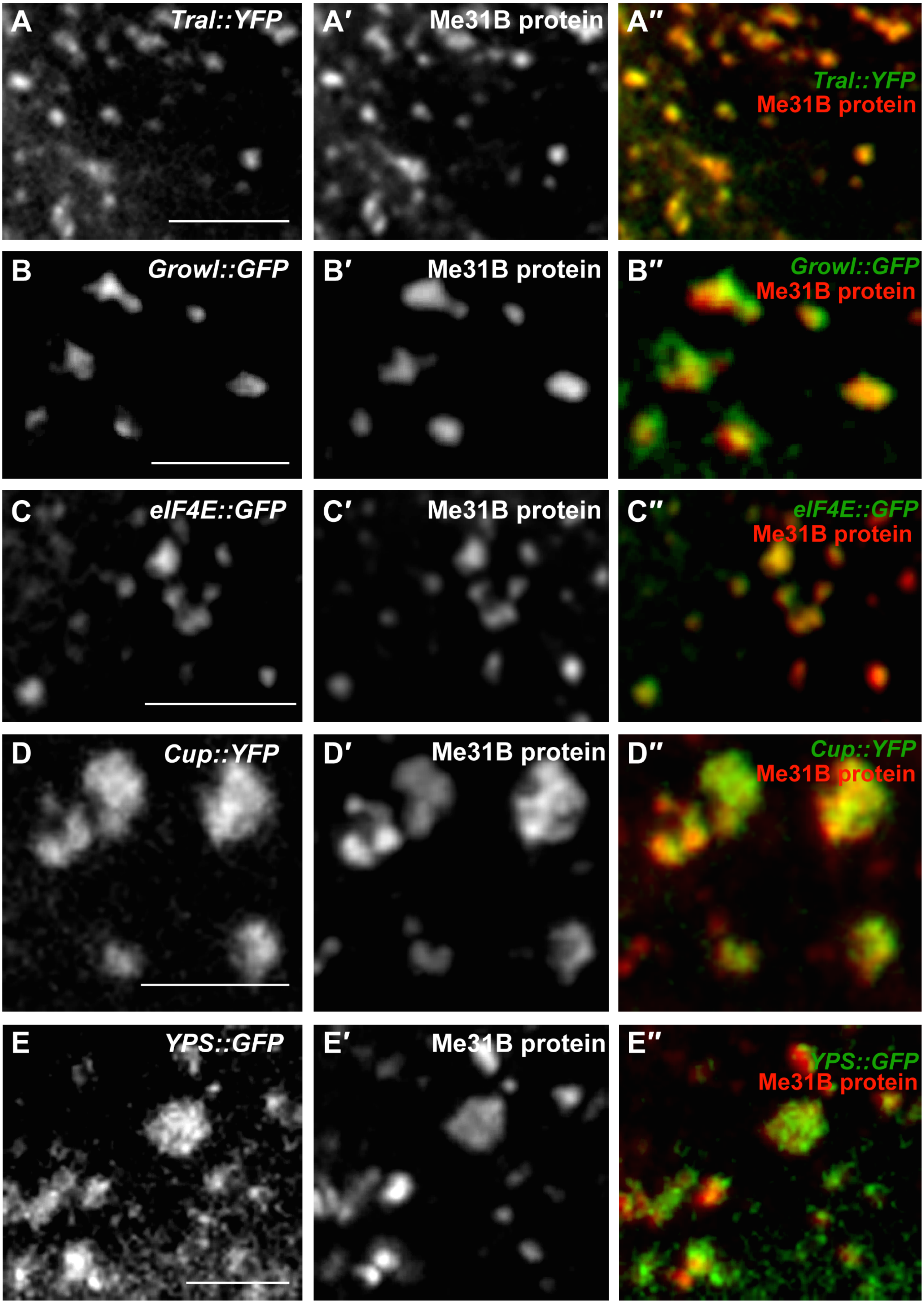
A
Tral::YFP
A′
Me31B protein
A″
Tral::YFP
Me31B protein
B
Growl::GFP
B′
Me31B protein
B″
Growl::GFP
Me31B protein
C
eIF4E::GFP
C′
Me31B protein
C″
eIF4E::GFP
Me31B protein
D
Cup::YFP
D′
Me31B protein
D″
Cup::YFP
Me31B protein
E
YPS::GFP
E′
Me31B protein
E″
YPS::GFP
Me31B protein

Figure S5. Nurse cell P bodies visualised by immunofluorescence have a similar protein complement to those in the oocyte, related to Figure 3

(A-E″) Protein detection by immunofluorescence on whole egg chambers. Tral (A), Growl (B), eIF4E (C), Cup (D), and YPS (E), labelled with fluorescent protein traps, colocalize extensively with Me31B antibody staining (A″,B″,D″,E″) in the nurse cell cytoplasm (n = 60). Note: some bodies are brighter in one channel, making a direct comparison difficult. All images were deconvolved and are 2 μm maximum intensity projections. Scale bars: 5 μm (A, C), 2.5 μm (B, D, E).
