# Supplementary material for: Localized Translation of gurken/TGF-α mRNA during Axis Specification Is Controlled by Access to Orb/CPEB on Processing Bodies
Source: Cell Rep. 2016 Mar 3;14(10):2451–62. doi: 10.1016/j.celrep.2016.02.038 (PMC4823467; doi:10.1016/j.celrep.2016.02.038)
Supplement: Document S2. Article plus Supplemental Information [file mmc2.pdf]

Cell Reports

Article

Localized Translation of *gurken/TGF-α* mRNA during Axis Specification Is Controlled by Access to Orb/CPEB on Processing Bodies

Graphical Abstract

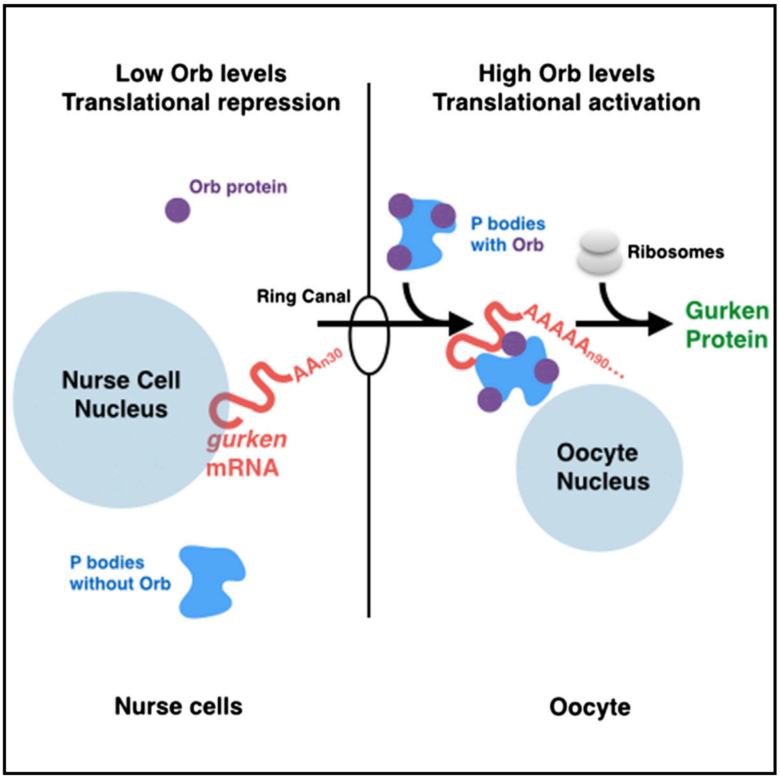

Authors

Alexander Davidson, Richard M. Parton, Catherine Rabouille, Timothy T. Weil, Ilan Davis

Correspondence

tw419@cam.ac.uk (T.T.W.),
ilan.davis@bioch.ox.ac.uk (I.D.)

In Brief

Localized transcripts that determine polarity are thought to be silenced during their transport by binding to translational repressors. Davidson et al. show that *gurken* mRNA, which sets up the primary *Drosophila* body axes, is instead regulated through lack of a translational activator, Orb, during its transport.

Highlights

- *gurken* mRNA is not silenced by known repressors during its transport
- In nurse cells, *gurken* mRNA is not associated with processing bodies
- In nurse cells, lack of sufficient Orb/CPEB silences *gurken* mRNA translation
- In oocytes, *gurken* mRNA is associated with Orb on processing bodies and translated

Davidson et al., 2016, Cell Reports *14*, 2451–2462
March 15, 2016 ©2016 The Authors
http://dx.doi.org/10.1016/j.celrep.2016.02.038

OPEN ACCESS
CellPress

Localized Translation of *gurken/TGF-α* mRNA during Axis Specification Is Controlled by Access to Orb/CPEB on Processing Bodies

Alexander Davidson,[1] Richard M. Parton,[1] Catherine Rabouille,[2,3] Timothy T. Weil,[4,*] and Ilan Davis[1,*]
[1]Department of Biochemistry, the University of Oxford, South Parks Road, Oxford OX1 3QU, UK
[2]Hubrecht Institute of the Royal Netherlands Academy of Arts and Sciences and University Medical Center Utrecht, Uppsalalaan 8, 3584 CT Utrecht, the Netherlands
[3]Department of Cell Biology, UMC Utrecht, Heidelberglaan 100, 3584 CX Utrecht, the Netherlands
[4]Department of Zoology, University of Cambridge, Downing Street, Cambridge CB2 3EJ, UK
*Correspondence: tw419@cam.ac.uk (T.T.W.), ilan.davis@bioch.ox.ac.uk (I.D.)
http://dx.doi.org/10.1016/j.celrep.2016.02.038
This is an open access article under the CC BY license (http://creativecommons.org/licenses/by/4.0/).

SUMMARY

In *Drosophila* oocytes, *gurken/TGF-α* mRNA is essential for establishing the future embryonic axes. *gurken* remains translationally silent during transport from its point of synthesis in nurse cells to its final destination in the oocyte, where it associates with the edge of processing bodies. Here we show that, in nurse cells, *gurken* is kept translationally silent by the lack of sufficient Orb/CPEB, its translational activator. Processing bodies in nurse cells have a similar protein complement and ultrastructure to those in the oocyte, but they markedly less Orb and do not associate with *gurken* mRNA. Ectopic expression of Orb in nurse cells at levels similar to the wild-type oocyte dorso-anterior corner at mid-oogenesis is sufficient to cause *gurken* mRNA to associate with processing bodies and translate prematurely. We propose that controlling the spatial distribution of translational activators is a fundamental mechanism for regulating localized translation.

INTRODUCTION

The regulation of translation in space and time is essential for a variety of physiological and developmental processes, such as axis specification in *Drosophila* and *Xenopus*, cell migration in fibroblasts, and synaptic plasticity in mammalian neurons (Medioni et al., 2012). Capped and polyadenylated mRNAs are by default translationally competent upon their export from the nucleus into the cytoplasm (Jackson et al., 2010). However, many mechanisms exist that can alter this default state and silence mRNA translation. Mechanisms include the binding to bona fide repressors (Richter and Lasko, 2011), denying access to ribosomes by inclusion in dense ribonucleoprotein (RNP) bodies (Weil et al., 2012), and preventing access to eIF4E by eIF4E binding proteins (Cao and Richter, 2002; Kamenska et al., 2014; Minshall et al., 2007; Nakamura et al., 2004; Richter and Sonenberg, 2005; Stebbins-Boaz et al., 1999; Wilhelm et al., 2003) and by having a reduced poly(A) tail length (Gamberi et al., 2002; Igreja and Izaurralde, 2011; Ivshina et al., 2014). Such translational control can be coupled to mRNA localization so that transcripts are translationally repressed while being transported and only activated when they reach their final destination. In this way, protein function can be targeted to specific subcellular locations with high fidelity.

In the *Drosophila* oocyte, the primary body axes are established through mRNA localization coupled to temporal and spatial regulation of the translation of *oskar* (*osk*), *bicoid* (*bcd*), *nanos* (*nos*), and *gurken* (*grk*) mRNA (Weil, 2014). All of these mRNAs are transcribed in the nuclei of the adjoining nurse cells before being deposited in the oocyte and localized. During their transport through the nurse cells and within the oocyte, such transcripts are thought to be maintained in a translationally silent state through a number of mechanisms, including those described above, followed by de-repression or activation at their final destination (Besse and Ephrussi, 2008; Richter and Lasko, 2011). However, it is not known whether the mechanisms of repression of each transcript are the same; nor is it clear how many mechanisms of repression are at play in each case.

Translational regulation of *osk* mRNA, which specifies the posterior of the future embryo and initiates the formation of the posterior germline, has formed the paradigm in the egg chamber for translational control through the binding of specific repressors. During the transport of *osk* mRNA, Bruno (Bru)/Arrest (Aret) binds to Bruno response elements (BREs) in its 3′ UTR. Together with polypyrimidine tract-binding protein (PTB), Bruno binding induces oligomerization of *osk* into translationally silenced particles that contain of up to 250 *osk* transcripts in the stage 10b oocyte (Besse et al., 2009; Chekulaeva et al., 2006; Kim-Ha et al., 1995; Little et al., 2015). BREs have been shown to act on *osk* mRNA in *trans*. Therefore, *osk* transcripts can confer Bruno-mediated repression to neighboring *osk* mRNAs within the same RNP (Hachet and Ephrussi, 2004; Reveal et al., 2010). This association breaks down when *osk* mRNA arrives at the oocyte posterior pole (Chekulaeva et al., 2006), allowing its translation. Furthermore, *osk* is subject to an

additional parallel mode of translational repression through the action of Cup, the *Drosophila* homolog of the mammalian eukaryotic initiation factor eIF4E binding protein 4E-transporter (4E-T) and functional homolog of *Xenopus* Maskin (Cao and Richter, 2002; Kamenska et al., 2014; Minshall et al., 2007; Nakamura et al., 2004; Nelson et al., 2004; Richter and Sonenberg, 2005; Stebbins-Boaz et al., 1999).

Cup represses *osk* mRNA in association with eIF4E and Bru by inhibiting recruitment of the small ribosomal subunit to the 5′ cap (Chekulaeva et al., 2006; Nakamura et al., 2004; Wilhelm et al., 2003). Moreover, Cup/Maskin/4E-T binds eIF4E and prevents it from associating with the translation initiation machinery (Cao and Richter, 2002; Kamenska et al., 2014; Minshall et al., 2007; Richter and Sonenberg, 2005; Stebbins-Boaz et al., 1999). Cup also works through repression of oo18 RNA binding protein (Orb), the *Drosophila* homolog of cytoplasmic polyadenylation element binding protein (CPEB) (Lantz et al., 1992; Wong and Schedl, 2011). Orb is required for the translational activation of *osk* mRNA by elongating its poly(A) tail (Chang et al., 1999; Castagnetti and Ephrussi, 2003; Juge et al., 2002), and high levels of Orb protein expression in the oocyte are ensured by the translational activation of *orb* mRNA by Orb protein (Tan et al., 2001). This feedback loop is controlled by the negative action of Cup, Ypsilon Schachtel (YPS), and *Drosophila* fragile X mental retardation (dFMR1) on *orb* translation (Costa et al., 2005; Mansfield et al., 2002; Wong and Schedl, 2011).

bcd mRNA is thought to be silenced in a similar manner as *osk*, but it utilizes a different translational repressor, Pumilio (Pum), which binds to conserved Nanos response elements (NREs) in the *bcd* 3′ UTR (Gamberi et al., 2002). Similarly, Glorund (Kalifa et al., 2006) and Smaug (Nelson et al., 2004; Zaessinger et al., 2006) bind to a translational control element (TCE) in the 3′ UTR of unlocalized *nos* mRNA to repress its translation (Crucs et al., 2000). During mid-oogenesis, our previous work has shown that localized *bcd* is translationally repressed in the core of processing bodies (P bodies), which consist of RNP complexes that are thought to regulate transcript stability and translation in a variety of systems (Decker and Parker, 2012; Weil et al., 2012). In the *Drosophila* oocyte, P bodies lack ribosomes and contain translational repressors, including the DEAD-box helicase maternal expression at 31B (Me31B) and Bru (Delanoue et al., 2007; Weil et al., 2012).

In contrast, there is less consensus regarding the mechanisms that are required for translational control of *grk* mRNA, particularly repression in nurse cells. Early in oogenesis, *grk* mRNA is localized and translated at the posterior of the oocyte, followed by a second phase of localization and localized expression at the dorso-anterior (DA) corner from mid-oogenesis. *grk* encodes a transforming growth factor α (TGF-α)-like signal that is secreted to the surrounding follicle cells to pattern dorsal cell fates (Neuman-Silberberg and Schüpbach, 1993). Dorso-ventral patterning also requires the heterogeneous nuclear RNP (hnRNP) Squid (Sqd), which has been shown to be necessary for correct Grk protein expression in the oocyte (Cáceres and Nilson, 2009; Clouse et al., 2008; Kelley, 1993; Li et al., 2014; Norvell et al., 1999). Although *grk* mRNA has been shown by biochemical analysis on ovaries (Norvell et al., 1999) to complex with Bruno through BRE-like sequences in its 3′ UTR, these match only weakly the BREs found in *osk* (Reveal et al., 2011). Furthermore, fluorescent expression reporters containing the BRE-like sequences from the *grk* 3′ UTR are subject to a low level of Bruno-mediated translational repression when compared with those containing *osk* BREs (Reveal et al., 2011).

We have previously established that, in the oocyte, *grk* mRNA is translationally repressed in transport particles and is then translated at its final destination in the DA corner of the oocyte, where it associates, in contrast to *bcd*, with the edge of P bodies (Weil et al., 2012). Importantly, the edge of P bodies has been shown to be decorated with ribosomes and enriched with Orb and Sqd (Clouse et al., 2008; Delanoue et al., 2007; Lantz et al., 1992; Li et al., 2014; Norvell et al., 1999; Weil et al., 2012). Interestingly, Orb has been shown to be required for the translation of *grk*, *osk*, and other localized mRNAs in the oocyte (Castagnetti and Ephrussi, 2003; Chang et al., 1999; 2001; Juge et al., 2002; Tan et al., 2001), and recently, Orb, together with Wispy (Wisp), a poly(A) polymerase, has been shown to be required for *grk* polyadenylation and Grk protein expression (Norvell et al., 2015). Indeed, phosphorylated, active Orb recruits Wispy and is required for the hyperadenylation and translation of *grk* (Norvell et al., 2015; Wong et al., 2011). However, it remains unclear when and where Orb acts in *grk* mRNA-localized expression in vivo and whether Orb association with P bodies is required to regulate *grk* translation.

Here, we address the mechanism in vivo by which translation of *grk* mRNA is prevented during its transport through nurse cells. We first tested the individual roles of previously suggested translational repressor proteins in the *Drosophila* egg chamber, including Me31B, Bru, and Sqd. We found that *grk* mRNA translational repression in nurse cells is not crucially dependent on any of these known repressors when tested individually, nor is *grk* present in the translationally silent core of P bodies in nurse cells. Instead, using immunofluorescence and electron microscopy, we found that wild-type nurse cell P bodies contain markedly lower levels of Orb compared with those in the oocyte. Increasing the levels of Orb protein within nurse cells by two independent methods causes *grk* mRNA to associate abnormally with nurse cell P bodies and also causes ectopic *grk* translation in nurse cells. Therefore, our data lead us to propose a model for spatial regulation of *grk* mRNA translation during *Drosophila* oogenesis in which *grk* transcripts are prevented from being translated in nurse cells by being denied access to sufficiently high levels of Orb, whereas, in the oocyte, *grk* is translated when it is anchored with Orb at the edge of P bodies.

RESULTS

grk Translational Silencing in Nurse Cells Is Not Dependent on Individual Translational Repressors as in *osk* Repression

Me31B and Bru are known to be crucial translational repressors of *osk* mRNA during its transport to the posterior of the oocyte because removal of each individually is sufficient to cause premature Osk protein expression (Chekulaeva et al., 2006; Nakamura et al., 2001, 2004; Wilhelm et al., 2003). We tested whether these regulators also individually repress *grk* mRNA translation during its transport. We first visualized the distribution of Grk

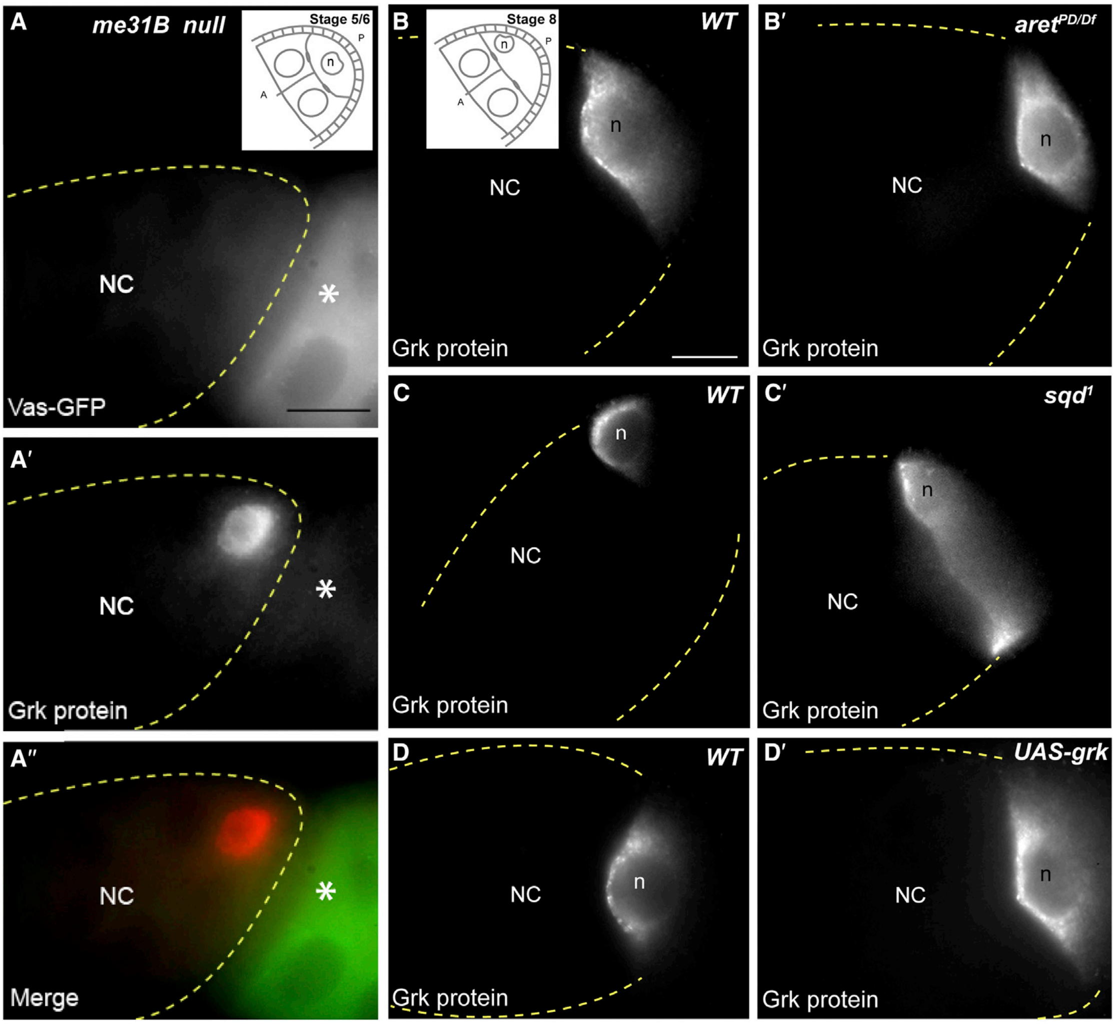

Figure 1. *grk* Does Not Require Translational Repressors to Maintain Translational Silencing in Nurse Cells

(A–A″) Homozygous Me31B-null germline clones generated by the flippase/flippase recognition target (FLP/FRT) system. (A) Loss of Me31B is marked by loss of Vasa-GFP fusion protein. Inset: schematic illustrating the relative position of the oocyte and nurse cells for a stage 5/6 egg chamber. (A′ and A″) Grk protein expression is restricted to the oocyte, with no staining in the nurse cells (n = 30). The asterisk marks a Vasa-GFP-positive egg chamber that is not an Me31B mutant. Egg chambers fail to develop to mid oogenesis in the *me31B* mutant background.

(B–D) In wild-type (WT) egg chambers, Grk protein expression is restricted to the oocyte, around the oocyte nucleus at mid-oogenesis (n = 30).

(B) Inset: schematic illustrating the relative position of the oocyte and nurse cells for a stage 8 egg chamber.

(B′) In weak *aret* mutants, the same pattern is observed (n = 60), with no ectopic staining in nurse cells. The same result is seen in medium and strong *aret* allelic combinations (Figures S1A and S1B).

(C′) In sqd^1 egg chambers, Grk protein is expressed along the anterior margin but not in nurse cells (n = 60).

(D′) In egg chambers overexpressing *grk* using the UAS-Gal4 system, Grk expression is restricted to the oocyte and is not expressed in nurse cells (n = 30) (Figure S2).

Scale bars, 15 μm. NC, nurse cell; Ooc, oocyte; n, oocyte nucleus. Dashed lines indicate the edges of the egg chamber.

protein in fly strains mutant for *me31B* using *me31B* heat shock-inducible germline clones (Nakamura et al., 2001). We found that, in *me31B*-null egg chambers, Grk protein is only expressed in the oocyte (Figure 1A), as in the wild-type. Similarly, Grk expression is unaffected in a number of allelic mutant combinations of *aret* (Bru mutant) (Yan and Macdonald, 2004; Figures 1B and 1B′; Figure S1). We also tested the role of Sqd, a heterogeneous nuclear RNA-binding protein, known to be required for *grk* mRNA anchoring (Delanoue et al., 2007) and translational repression in the oocyte (Cáceres and Nilson, 2009; Clouse et al., 2008; Li et al., 2014; Norvell et al., 1999). In sqd^1 mutant oocytes, Grk protein is ectopically expressed along the entire anterior margin, resulting is a dorsalized egg (Figure 1C versus Figure 1C′) (Kelley, 1993; Norvell et al., 1999). However, we found that Grk protein is not expressed in nurse cells of sqd^1 mutant egg chambers, showing that Sqd is not required for repressing *grk* translation in nurse cells (Figure 1C′). Collectively, we conclude that, unlike *osk*, none of the factors we tested

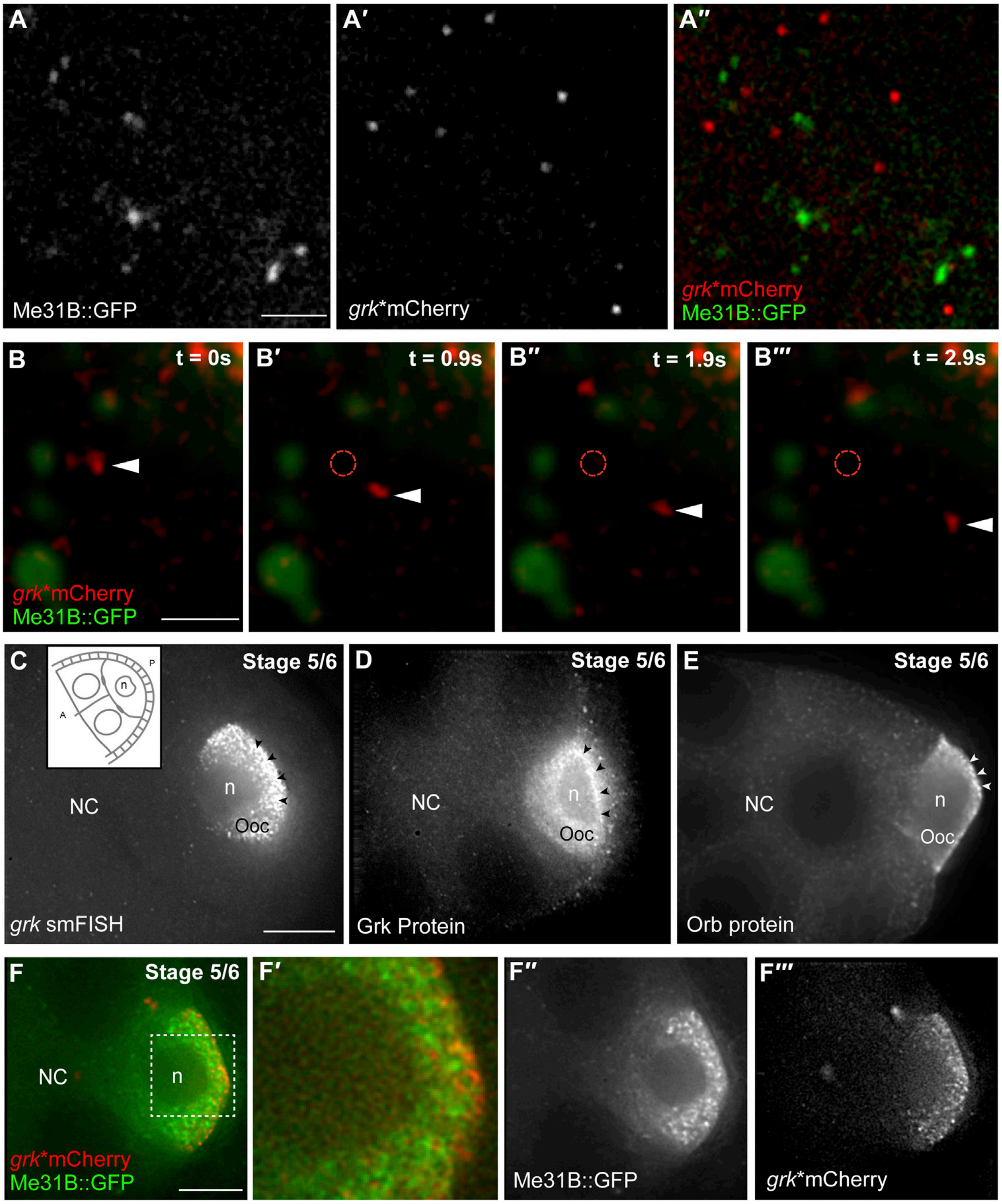

Figure 2. *grk* mRNA Is Not Associated with P Bodies in Nurse Cells

(A–A′′) In egg chambers expressing Me31B::GFP, P bodies rarely associate (16% of particles, n = 297) with *grk* mRNA particles labeled with single-molecule FISH in the nurse cell cytoplasm. (A) Me31B::GFP labeling P bodies, (A′) *grk* mRNA particles labeled with single-molecule FISH, and (A′′′) overlay of Me31B::GFP labeling P bodies in green and single-molecule FISH labeling *grk* mRNA in red.

(B–B′′′) Consecutive time points in a time-lapse series of a live egg chamber expressing *grk*mCherry* and Me31B::GFP. *grk* particles (arrowheads) move independently of Me31B assemblies in the nurse cell cytoplasm (n = 89). Dashed red circles indicate the positions of *grk* particles at t = 0 s.

(C–E) In early oogenesis, *grk* localized to the oocyte posterior where it is locally translated.

(C) *grk* smFISH on an early-stage egg chamber showing the localization of *grk* at the posterior (arrowheads) (n = 5). Inset: schematic illustrating the relative position of the oocyte and nurse cells for a stage 5/6 egg chamber.

(D) Anti-Grk antibody labeling showing the gradient in Grk protein from a local enrichment at the posterior (arrowheads) (n = 5).

(E) Anti-Orb antibody labeling showing increased Orb in the oocyte (arrowheads) (n = 5).

(legend continued on next page)

individually repress *grk* mRNA translation during its transport in nurse cells.

Ectopically expressing *osk* mRNA leads to ectopic Osk protein expression in nurse cells, suggesting that the *osk* repression machinery is saturated by an excess of *osk* mRNA (Snee and Macdonald, 2004). To test whether a similar saturable mechanism exists to repress *grk* mRNA translation, we ectopically expressed *grk* mRNA to saturate any putative repression machinery. We used the UAS-Gal4 system to overexpress full-length *grk* transcripts (Bökel et al., 2006; Weil et al., 2012) at an average of 3-fold the level in wild-type nurse cells (Figure S2). This results in ectopic Grk expression, but only along the anterior margin of the oocyte, not in nurse cells (Figure 1D versus Figure 1D′). These data strengthen the notion that, in contrast to *osk*, *grk* translation in nurse cells is not mediated through the saturable binding of repressors.

grk mRNA Associates Differently with P Bodies in Nurse Cells and Oocytes

We next tested whether *grk* is maintained in a translationally silent state in nurse cells by localization to the ribosome-depleted and translationally silent core of P bodies in a similar manner to *bcd* in the oocyte (Weil et al., 2012). We visualized *grk* mRNA in nurse cells with single-molecule fluorescence in situ hybridization (smFISH) (Little et al., 2015) of egg chambers expressing *Me31B::GFP*, a canonical marker for P bodies in the oocyte (Weil et al., 2012). We found that small foci of *grk* mRNA, similar but weaker in intensity than oocyte transport particles (Figure S2A and S2A′), are evenly distributed in the nurse cell cytoplasm, with only a minority associated with the edge of nurse cell P bodies (Figures 2A and 2A″).

To test whether *grk* associates with P bodies in nurse cells at mid-oogenesis, we co-visualized *grk* in live nurse cells with the MS2-MCP system (*grk*mCherry*) (Bertrand et al., 1998; Forrest and Gavis, 2003; Jaramillo et al., 2008) and a number of proteins labeled by fluorescent protein traps (Buszczak et al., 2007). We found that *grk*mCherry* particles do not move with Me31B in nurse cells and transiently associate with P bodies at a significantly lower frequency (8%; n = 486) than in the oocyte (41%) (Weil et al., 2012; Figures 2B–2B‴). These data suggest that, within our detection limits, *grk* mRNA does not move with P body proteins and is not localized to the interior of P bodies. We also tested whether the same was true at early stages by assessing the distribution of *grk* mRNA, Grk protein in fixed material (Figures 2C and 2D), and the interaction of *grk* mRNA with P bodies in live material (Figures 2F–2F‴). We found that, in early stages, before the oocyte nucleus migrates, *grk* mRNA particles are dynamic all over the egg chamber, except when they are associated with P bodies at the posterior of the oocyte (Figure 2F′), where Orb is enriched (Figure 2E). We also found that Grk protein was enriched at the posterior of the oocyte and present at lower levels in other parts of the oocyte in a gradient that is consistent with diffusion away from its site of translation at the posterior (Figure 2D). Considering all of our data, we conclude that *grk* mRNA is likely to be translated at its site of anchoring in both early and late stages of *grk* mRNA localization. Moreover, *grk* mRNA is unlikely to be repressed by association with known saturable translational repressors either in the nurse cell cytoplasm or within P bodies.

The Translational Activator Orb Is Largely Depleted in Nurse Cells

Although we cannot completely eliminate the possibility that *grk* translation is controlled by a redundant and/or yet to be identified repression mechanism, our data prompted us to examine known translation activators such as Orb. To address this, we first re-characterized *orb*^{mel} mutants that expresses a truncated version of *orb* mRNA, resulting in lower levels of Orb protein from stage 7 of oogenesis onward (Christerson and McKearin, 1994). We observed a loss of Grk expression in *orb*^{mel} mutants (Figure S3), in agreement with previously published data (Chang et al., 2001). To begin to understand why *grk* mRNA is translated in wild-type oocytes but not in the nurse cells, we characterized the distribution of Orb protein in P bodies in the two compartments using immuno-electron microscopy (IEM) on ultrathin frozen sections of *Drosophila* egg chambers (Figure 3; Figure S4). We found that the level of Orb protein in nurse cell P bodies is 18 times lower than in oocyte P bodies (n = 10 P bodies; Figure 3D versus Figure 3D′). Using immuno-fluorescence detection on fixed egg chambers, we confirmed that the overall level of Orb is much lower in nurse cells compared with oocytes (Figures 3E, 4A, and 4C).

We then tested whether the overall composition of the nurse cell P bodies was different from those in the oocytes. We found that the key P body markers Me31B (Figure 3A) and Bru (Figure 3B) have a similar enrichment in nurse cells and the oocyte and that ribosomes are excluded from the nurse cell P bodies as they are from P bodies in the oocyte (Figure 3C). We also visualized P body protein composition by immunofluorescence and found that Me31B co-localizes with the canonical P body components Trailerhitch (Tral), Growl, eIF4E, Cup, and YPS in P bodies in nurse cells as they do in the oocyte (Figure S5). We conclude that the difference in Orb protein content of the P bodies in the two tissues is specific and that Orb is relatively depleted from nurse cell P bodies compared with those in the oocyte.

Orb Is a Key Determinant for *grk* Translation in Nurse Cells

To test whether low abundance of Orb in nurse cells is the key factor that prevents *grk* mRNA translation, we used the UAS-Gal4 system to drive the level of Orb in nurse cells to a similar level as in the wild-type oocyte (*UASp-orb* and *TubulinGal4VP16*) (Li et al., 2014). This approach also results in

(F–F‴) Early-stage living egg chamber expressing *grk*mCherry* and Me31B::GFP showing local docking of *grk* on Me31B-labeled P bodies at the posterior, taken from a time-lapse series (n = 4). (F) Composite image showing the interdigitation of *grk* with Me31B at the posterior. (F′) Enlargement of the region identified by the dashed box in (F). (F″) Me31B::GFP showing the decreasing gradient in P body density from posterior to anterior. (F‴) *grk*mCherry* showing the locally docked *grk* mRNA at the posterior.
Scale bars, 2 μm (A and B) and 10 μm (C and F).

OPEN
ACCESS
CellPress

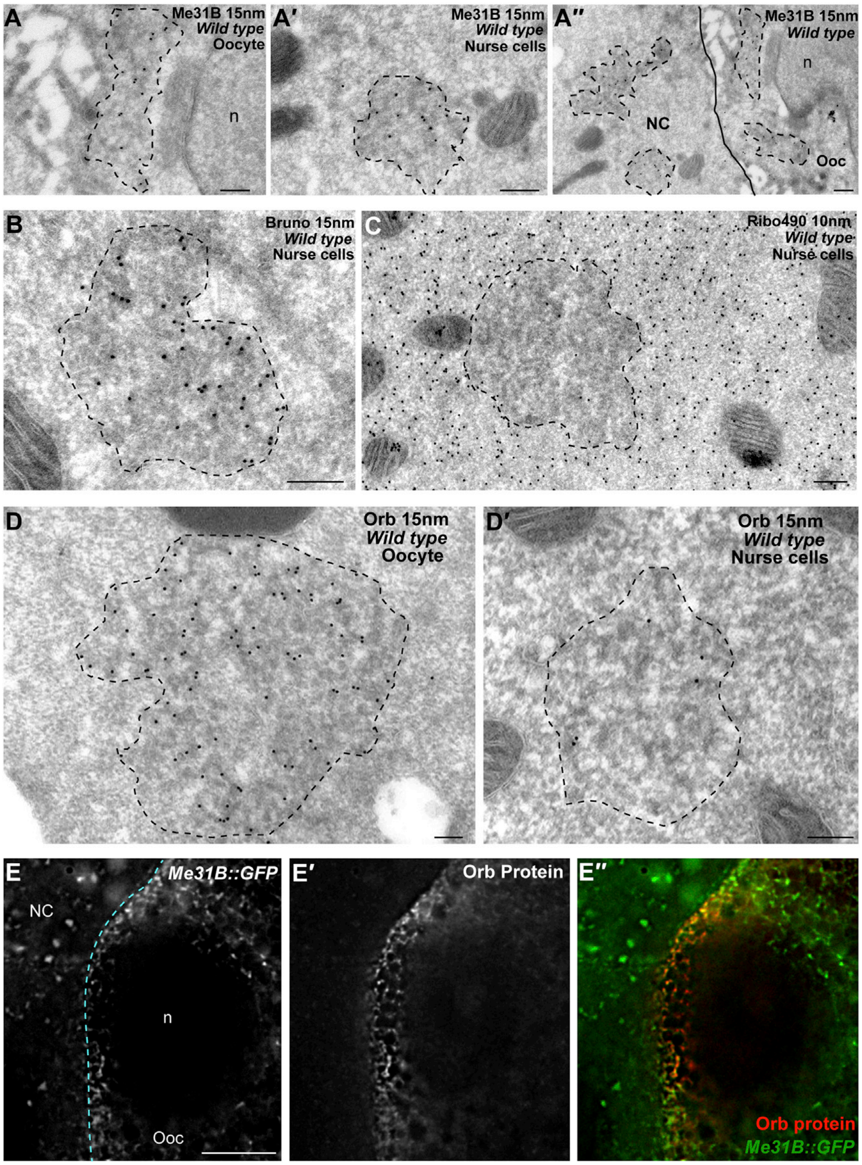

Figure 3. P Bodies in Nurse Cells Contain Similar Proteins as those in the Oocyte but Have Significantly Lower Levels of Orb

(A–D′) Protein detection by IEM on ultrathin frozen sections of nurse cells. Dashed black lines mark the edge of P bodies.

(A–A″) Anti-Me31B (10 nm) is highly enriched in the core of P bodies in the oocyte (A) and nurse cells (A′). A″ is a lower-magnification image showing P bodies containing Me31B in both nurse cells and the oocyte. The solid black line marks the boundary between the nurse cells and the oocyte.

(B) Anti-Bru (15 nm) is highly enriched in the P body core and in P bodies at the dorso-anterior corner of the oocyte. Proteins found at the core of P bodies in the oocyte are also detected in nurse cell P bodies by immunofluorescence (Figure S5). The cyan line marks the boundary between the nurse cells and the oocyte.

(C) Anti-ribo 490 (10 nm) shows ribosomes predominantly excluded from P bodies in nurse cells.

(D and D′) Anti-Orb (15 nm) is present in P bodies in the oocyte and is enriched at the edge (D, see also Figure S4), but P bodies in nurse cells contain significantly lower levels of Orb (D′).

(E–E″) Nurse cell-oocyte boundary of the DA corner of an egg chamber expressing Me31B::GFP (E) stained with anti-Orb (E′) (n = 30). P bodies in the nurse cell cytoplasm express significantly lower levels of Orb compared with in the oocyte. (E) Me31B::GFP labeling P bodies, (E′) Anti-Orb labeling Orb protein, and (E‴) overlay of Me31B::GFP labeling P bodies in green and anti-Orb labeling Orb protein in red.

Scale bars, 200 nm (A–A″, B, C, D, and D′) and 10 μm (E).

a higher level of Orb protein in the oocyte of these egg chambers (Figure 4A versus Figure 4A′). Importantly, in these egg chambers, Grk protein is ectopically expressed in nurse cells in a highly reproducible pattern along nurse cell boundaries (Figure 4B versus Figure 4B′), as expected for a secreted protein that is normally trafficked into the overlying follicle cells when expressed at the DA corner (Queenan et al., 1999). We conclude that the ectopic expression of Orb in nurse cells drives the premature translation of *grk* mRNA in nurse cells. We propose that the absence of *grk* translation in wild-type nurse cells is due to the low level of Orb in this tissue.

Previous work has shown that *orb* transcripts can be detected in nurse cells as well as in the oocyte (Wong and Schedl, 2011) but that *orb* translation is repressed by Cup in nurse cells (Wong and Schedl, 2011). To test whether Cup is required to suppress the translation of *orb* mRNA in nurse cells and thus prevent *grk* translation, we stained *cup* mutant egg chambers with antibodies against Grk. We used cup^{1355}, in which a P element insertion into the untranslated exon 1 causes a reduction in the level of Cup protein expression (Karpen and Spradling, 1992). As expected, we found an upregulation of Orb in cup^{1355} mutant nurse cells (Figure 4C versus Figure 4C′; n = 238), and, strikingly, we found that Grk protein is expressed in nurse cells and enriched along cell-cell boundaries, as in *UAS-orb* egg chambers (Figure 4D versus Figure 4D′). Interestingly, we found that Grk protein expression is stronger in cup^{1355} than *UAS-orb* egg chambers. This is consistent with Cup partly repressing excess Orb in *UAS-orb* egg chambers, whereas, in cup^{1355}, *orb* translation is fully derepressed because of the lack of Cup. These results show that, when Cup-mediated repression of *orb* mRNA is absent in egg chambers, Orb is upregulated in nurse cells and *grk* is translated.

grk Is Translationally Activated by Ectopic Orb on the Edge of Nurse Cell P Bodies

At the DA corner of the oocyte, and most likely also at the posterior earlier in oogenesis, *grk* is translated when it docks at the

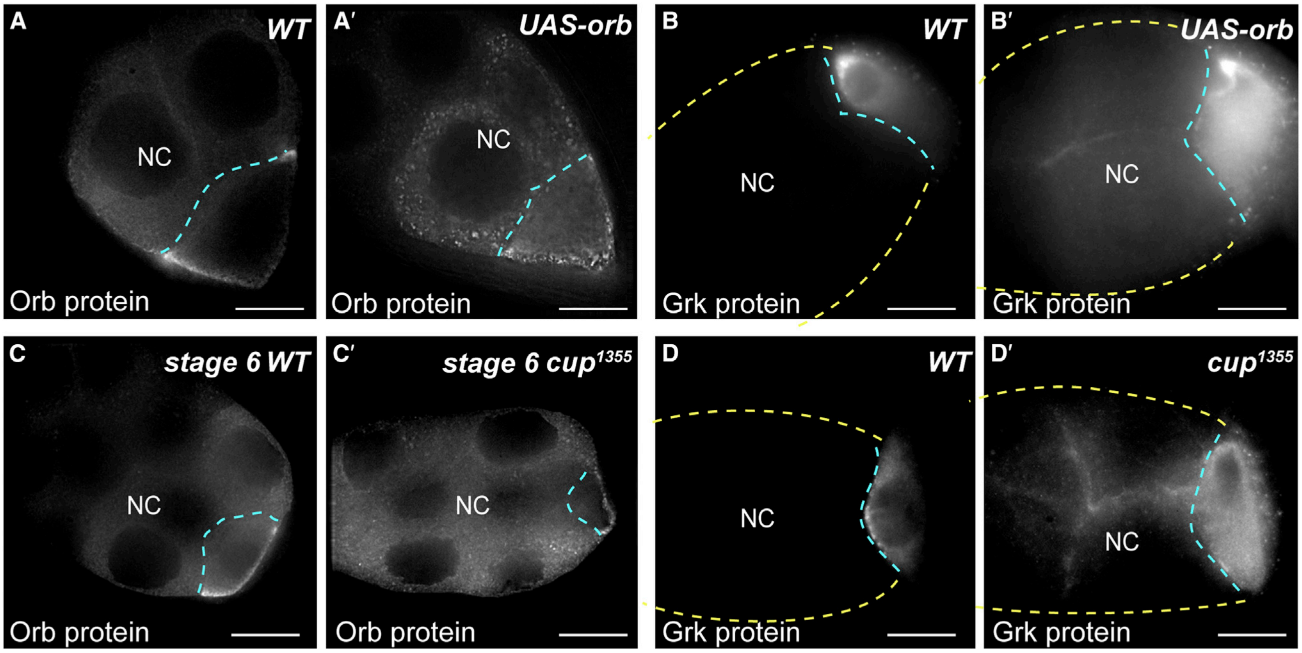

Figure 4. When Orb Is Upregulated, *grk* Is Ectopically Translated in Nurse Cells

(A and A′) Antibody staining using anti-Orb. In WT egg chambers, Orb is expressed at significantly higher levels in the oocyte than in the nurse cells (A) (n = 258). In egg chambers overexpressing orb using the UAS-Gal4 system, Orb is overexpressed in both the nurse cells and the oocyte in puncta (A′) (n = 60).

(B and B′) Antibody staining using anti-Grk. In WT egg chambers, Grk expression is restricted to the oocyte and is localized to the dorso-anterior corner (B) (n = 300). In egg chambers overexpressing orb using the UAS-Gal4 system, Grk is expressed both throughout the oocyte and also in nurse cells along cell-cell boundaries (B′) (n = 60).

(C and C′) Antibody staining using anti-Orb of a stage 6 egg chamber. In WT egg chambers, Orb is expressed at significantly higher levels than in nurse cells (C). In *cup*^{1355} mutant egg chambers, Orb is overexpressed in nurse cells compared with the oocyte (C′) (n = 238).

(D and D′) Antibody staining using anti-Grk. In WT egg chambers Grk expression is restricted to the oocyte and is localized to the dorso-anterior corner (D) (n = 300). In cup^{1355} mutant egg chambers, Grk is expressed in nurse cells along cell-cell boundaries (D′) (n = 274). The staining in nurse cells is noticeably stronger than in egg chambers overexpressing Orb using the UAS-Gal4 system.

Scale bars, 15 μm. Dashed yellow lines indicate the edges of the egg chamber, and dashed cyan lines delineate the oocyte boundary.

Orb-enriched edge of P bodies (Figures 2, 3, 4A, and 4C; Weil et al., 2012). If the key functional difference between P bodies in the oocyte and nurse cells is their level of associated Orb, then one would expect to find Orb at the edge of the nurse cell P bodies when it is ectopically expressed there. To test this prediction, we first examined Orb and Me31B distribution in the nurse cells of *UAS-orb* egg chambers and found that they colocalize (Figure 5A). Similarly, we found that Orb co-localizes with Tral in the nurse cells of *cup*^{1355} mutant egg chambers (Figure 5B). To test whether ectopic Orb is enriched at the edge of nurse cell P bodies as it is in the oocyte, we performed 3D structured illumination microscopy (3D-SIM) (Schermelleh et al., 2008) on the OMX microscope (Dobbie et al., 2011). 3D-SIM reveals Orb puncta present at the edge of the Me31B-labeled P bodies in nurse cells (Figures 5C–5C″). We conclude that ectopic Orb in nurse cells is enriched at the edge of P bodies as it is at the DA corner of the oocyte.

To test whether ectopically expressing Orb in nurse cells targets *grk* to the edge of P bodies, allowing its translation, we performed smFISH for *grk*. As mentioned above, we found that, in nurse cells of wild-type egg chambers, *grk* seldom colocalizes with P bodies (Figures 2A and 5D–5D″). Conversely, in egg chambers in which Orb is ectopically expressed in nurse cells, *grk* foci colocalize 3-fold more with P bodies compared with the wild-type (Figures 5E–5E″). We conclude that overexpressed ectopic Orb associates with P bodies anchoring *grk* transcripts to activate translation. Therefore, we propose that *grk* is not translated in the nurse cells of wild-type egg chambers because, unlike in the oocyte, Orb is not present in nurse cells at the edge of P bodies.

DISCUSSION

Our results show that the translational regulation of *grk* in nurse cells, where the transcript is synthesized, occurs by a different mechanism from that of *osk* and *bcd* mRNA in the oocyte. *osk* is primarily translationally regulated by binding to individually essential translational repressors (Nakamura et al., 2001, 2004; Chekulaeva et al., 2006), whereas *bcd* is translationally repressed through its inclusion in the ribosome-depleted interior of P bodies (Weil et al., 2012). In contrast, our results show that translational silencing of *grk* mRNA during its transport in nurse cells is not affected when the repressors affecting *osk* are individually removed and that it is not localized within P bodies while being transported and repressed. These observations highlight a

OPEN
ACCESS
CellPress

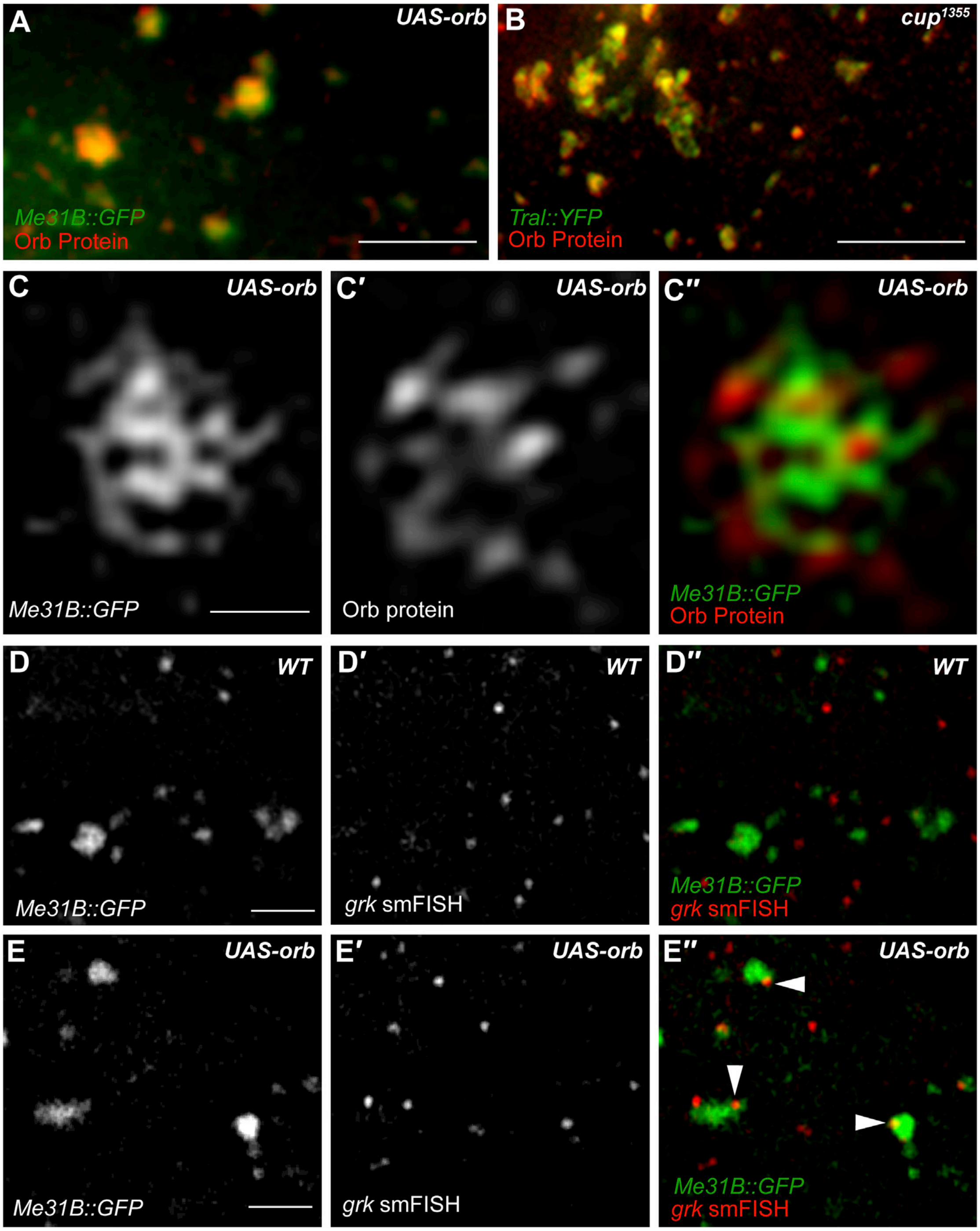

Figure 5. When Orb Is Upregulated, It Is Expressed at the Edge of Nurse Cell P Bodies, and *grk* Is Targeted to P Bodies in Nurse Cells

(A) Nurse cell cytoplasm of an egg chamber expressing *Me31B::GFP* and overexpressing *orb* using the UAS-Gal4 system, stained with anti-Orb. Orb and Me31B co-localize in P bodies in the nurse cell cytoplasm. The image is a 4-μm average intensity projection (n = 30).

(B) Nurse cell cytoplasm of a *cup*^{1355} mutant egg chamber that is also expressing *Tral::YFP*, stained with anti-Orb. Orb and Tral co-localize in P bodies in the nurse cell cytoplasm (n = 30). The image is a 4-μm average intensity projection.

(C–C″) 3D-SIM of the nurse cell cytoplasm of an egg chamber expressing *Me31B::GFP* and overexpressing *orb* using the UAS-Gal4 system, stained with anti-Orb. 3D-SIM resolves Orb puncta, which are enriched at the edge of the Me31B-labeled P body that has a reticulated structure, as shown previously (Weil et al., 2012). (C) Me31B::GFP labeling P bodies, (C′) anti-Orb labeling Orb protein, and (C″) overlay of Me31B::GFP labeling P bodies in green and anti-Orb labeling Orb protein in red.

(legend continued on next page)

OPEN ACCESS
CellPress

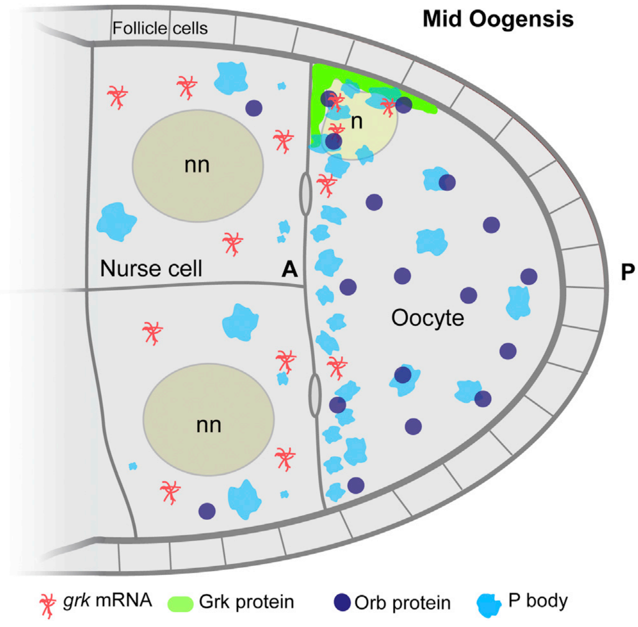

Figure 6. *grk* Is Translationally Silenced in Nurse Cells by Being Denied Access to Its Translational Activator, Orb

A model of *grk* translational regulation by restricted spatial access to Orb. In the nurse cells of *WT* egg chambers, P bodies lack in Orb. *grk* mRNA does not dock with P bodies and is not translated. At the oocyte DA corner, Orb is enriched at the edge of P bodies where *grk* mRNA docks. *grk* is translated and then secreted to the follicle cells around the oocyte nucleus.

major difference in the mechanisms of translational repression between *grk, bcd*, and *osk*.

Although the translational activation of *grk* mRNA at its final destination in the oocyte has been shown to require the polyadenylation factor and activator Orb (Chang et al., 2001; Norvell et al., 2015), probably at the edge of P bodies (Weil et al., 2012), its role during the transport of *grk* in nurse cells has not been previously addressed. Our data show that the relative depletion of Orb from nurse cells compared with the oocyte is sufficient to prevent *grk* mRNA from being translated in nurse cells. We further show that P bodies are present in wild-type nurse cells and that they have the same apparent composition and ultrastructure as in the oocyte, except that they lack Orb and *grk* mRNA. Interestingly, when Orb expression is driven in nurse cells to similar levels as occur in the wild-type oocyte using UAS-Orb, we find that Orb and *grk* mRNA are associated with nurse cell P bodies, leading to *grk* mRNA premature translation in nurse cells. These results suggest that the absence of Orb in nurse cells is the limiting factor that prevents *grk* translation before it arrives in the oocyte.

We also obtained similar results using a *cup* mutant in which Orb levels are higher in nurse cells. Why cup normally represses Orb expression only in nurse cells is unclear, but we nevertheless found that *grk* is prematurely translated in nurse cells of *cup* mutants. Based on our results above and previously published work (Wong and Schedl, 2011), we favor the simplest interpretation: that, in *cup* mutants, Orb expression is elevated sufficiently to allow *grk* translation. However, we cannot completely exclude the possibility that Cup could also be involved directly in repressing *grk* translation in nurse cells through Cup's known role in excluding eIF4E in the case of other transcripts (Nakamura et al., 2004; Wilhelm et al., 2003). Whether Cup acts by influencing Orb alone or also acts directly on *grk* mRNA translation, Cup and Orb spatially regulate translation of *grk* mRNA in egg chambers. It is interesting to note that, in *Xenopus,* CPEB activation of the translation of mRNA in the oocyte is temporally rather than spatially regulated through the action of hormone signals (Sarkissian et al., 2004), leading to activation of translation at precisely orchestrated times by lengthening of poly(A) tails (Hake and Richter, 1994).

Consistent with the published literature and its canonical function, Orb most likely acts on *grk* through cytoplasmic polyadenylation by binding to a polyadenylation element at the 3′ end of *grk* mRNA near the polyadenylation hexanucleotide signal (AAUAAA) (Chang et al., 1999; Fox et al., 1989; Kim and Richter, 2006; Norvell et al., 2015; Tan et al., 2001). Such an activity for Orb protein was first established for its homolog in *Xenopus*, CPEB (Fox et al., 1989; Hake and Richter, 1994). Orb is also known to bind *osk* and *k(10)* mRNAs and control their translation by modulation of poly(A) tail length (Castagnetti and Ephrussi, 2003; Chang et al., 1999, 2001; Juge et al., 2002; Tan et al., 2001; Wong and Schedl, 2011). Although there is no direct evidence that Orb binds *grk* mRNA, recent work shows that Orb and Wispy cooperate to polyadenylate localized *grk* mRNA in egg chambers (Chang et al., 2001; Norvell et al., 2015). It is certainly possible to imagine alternative models for how Orb acts on *grk*, such as polyadenylating and promoting the translation of other translational activators of *grk*. However, in the absence of any further direct evidence for such alternative mechanisms, we favor the simpler interpretation that Orb acts directly on *grk* by polyadenylating it and activating its translation.

Considering all of our data in the context of previously published work, we propose the following model for translational regulation of *grk* mRNA. While being transported in the nurse cell cytoplasm, *grk* mRNA is not translated because it fails to associate with Orb at the edge of P bodies. Only when *grk* enters the oocyte and moves to the dorso-anterior corner, where Orb levels are highest, does it become associated with Orb on the edge of P bodies, causing its localized translational activation (Figure 6).

Previous work indicates that *grk* translation is restricted to the posterior in early oocytes and at the DA corner at mid-oogenesis (Chang et al., 2001; Neuman-Silberberg and Schüpbach, 1993, 1994). More recent work suggests that this restricted translation is due to localization of active,

(D) In OrR egg chambers, *grk* particles in nurse cells (D′) rarely colocalize with P bodies (D′′) in nurse cells (16% of particles, n = 297).
(E) In *UAS-orb* egg chambers, *grk* particles in nurse cells (E′) colocalize with P bodies (E) (53% of particles, n = 395) and seem to be docked at the P body edge (E′′).
Scale bars, 2 μm (A, B, and D–E′′) and 1 μm (C–C′′).

phosphorylated Orb (Norvell et al., 2015; Wong and Schedl, 2011). This may provide a plausible explanation for why *grk* mRNA does not associate with P bodies containing Orb in the middle of the oocyte; namely, if Orb is only phosphorylated and active in the DA corner. However, we cannot exclude the possibility that other factors, such as PABP55B, Encore (Enc), and Sqd, could also be required in the oocyte for localized *grk* translation (Clouse et al., 2008; Hawkins et al., 1996, 1997; Van Buskirk et al., 2000). Certainly, Sqd protein has been shown to bind *grk* mRNA directly and to regulate its translation (Li et al., 2014; McDermott et al., 2012; Norvell et al., 1999), possibly through anchoring, because previous work has also shown that, in the absence of Sqd protein, *grk* mRNA fails to anchor at the oocyte DA corner and is ectopically translated along the anterior (Cáceres and Nilson, 2009; Delanoue et al., 2007; Jaramillo et al., 2008; Norvell et al., 1999). Our data show that Sqd does not repress *grk* mRNA translation in nurse cells, leading us to interpret the function of Sqd in repression of *grk*, identified by previous biochemical work (Li et al., 2014), as occurring in the oocyte and not in nurse cells.

The well studied localized mRNAs *osk*, *bcd*, *grk*, and *nos* each have distinct profiles of translational regulation in time and space. For example, *osk* and *grk* are transported in a repressed state and are translationally activated when they arrive at their final destinations in the oocyte (Ephrussi et al., 1991; Neuman-Silberberg and Schüpbach, 1993), whereas localized *bcd* remains repressed within P bodies until egg activation (Weil et al., 2012). Our results suggest that spatial regulation of translation can be achieved by restricting the levels of a single activator, Orb. We propose that this could be a widespread mechanism of preventing translation of localized transcripts while they are being transported to their final destination.

EXPERIMENTAL PROCEDURES

Fly Strains

Stocks were raised on standard cornmeal-agar medium at 25°C. The wild-type was Oregon R (OrR). Mutant lines were as follows: *hsFLP/w ; me31B*$^{\Delta1}$ *FRT40A, hsFLP/w ; me31B*$^{\Delta2}$ *FRT40A* (Nakamura et al., 2001); *aret*^{PD}, *aret*^{PA}, and *aret*^{QB} (Schüpbach and Wieschaus, 1991); *squid*^{1} (Kelley, 1993); and *cup*^{1355} (Karpen and Spradling, 1992). The heat shock marker line was *hsFLP/w ; gfp-vas FRT40A*. *MS2-MCP(FP)* lines were as follows: *grk -(MS2)*^{12} (Jaramillo et al., 2008) and *Pnos-NLS-MCP-mCherry* (Weil et al., 2012). P body markers were as follows: *Me31B::GFP* (Buszczak et al., 2007) (CG4916), *Tral::YFP* (D. St Johnston CG10686), *Growl::GFP* (Buszczak et al., 2007) (CG14648), *eIF-4E::GFP* (Buszczak et al., 2007) (CG4035), *Cup::YFP* (D. St Johnston, CG11181) *YPS::GFP* (Buszczak et al., 2007) (ZCL1503). Overexpression lines were as follows: maternal tubulin driver TubulinGal4-VP16; *UASp grk3A* based on genomic sequence DS02110, which includes the full 3′ and 5′ UTRs (Bökel et al., 2006), and *UASp-orb* (Li et al., 2014). The deficiency line was *Df(2L)esc-P2-0* (Bloomington, BL3130). For Me31B germline clones, the heat shock regime was performed as described previously (Nakamura et al., 2001).

Electron Microscopy Sample Preparation and Analysis

Protein detection was performed by IEM as described previously (Delanoue et al., 2007; Herpers et al., 2010; Weil et al., 2012).

Antibodies

The antibodies used were Grk, mouse monoclonal (Developmental Studies Hybridoma Bank [DSHB], 1D-12, 1:300), Orb 4H8 (DSHB, 1:30), Me31B (a gift from A. Nakamura, 1:1000), Bruno (a gift from A. Ephrussi, 1:300), and α ribo-490 (a gift from J. Van Minnen, 1:300).

Fluorescence Imaging

Flies were prepared and ovaries dissected and mounted for imaging according to standard protocols (Delanoue et al., 2007; Parton et al., 2011; Weil et al., 2012). Unless otherwise stated, the egg chambers shown in the figures were mid-oogenesis (stages 7–9). Imaging was performed on a DeltaVision CORE wide-field deconvolution system (Applied Precision, a subsidiary of GE Healthcare) based on an Olympus IX71 microscope using ×20 0.75 numerical aperture (NA) dry, ×100 1.4 NA oil, and ×100 1.3 NA silicon oil objectives, a 16-bit Roper Cascade II camera, and standard Chroma filter sets. Where required, images were deconvolved with the SoftWoRx Resolve 3D constrained iterative deconvolution algorithm (Applied Precision). For live-cell imaging, *grk* mRNA particles were imaged close to the nurse cell nuclei at a single shallow plane of focus. Exposures of 300 ms at 3 frames/second were taken to achieve the optimum balance between signal-to-noise and temporal resolution for deconvolution and particle tracking. Analysis of fluorescence intensity in Orb antibody-stained egg chambers was performed using FIJI (V1.0, http://fiji.sc/wiki/index.php/Fiji). 3D-SIM imaging was performed on the OMX V3 microscope (GE Healthcare) as described previously (Weil et al., 2012).

Immunofluorescence on Fixed *Drosophila* Oocytes

Adult females flies were fattened as described above. An optimized fixation and staining protocol was then used to reduce possible antibody penetration artifacts that can be associated with immunofluorescence. Flies were dissected directly into freshly made 4% paraformaldehyde in PBST (0.1%) (paraformaldehyde stock solution: 16% methanol-free, ultrapure EM grade, Polysciences). Ovarioles were splayed using tweezers and a probe (Fine Science Tools) but not fully separated. Individual ovaries were transferred into an Eppendorf tube, and then 800 μl heptane was added before mixing briefly by vortex. Ovaries were fixed for no more than 15 min in total, followed by three rinses and three washes of 10 min in PBST. Following PBST washes, ovaries were washed for 5 min in PBS with Triton X-100 (PBTX, 0.01%) and then rinsed in PBST. Ovaries were blocked in 4% BSA in PBST for 30 min. Primary antibody was added at the required concentration in PBST for 2 hr at room temperature, followed by three rinses and three washes of 20 min in PBST. Secondary antibody was added at 1:500 in PBST for 1 h at room temperature, followed by three rinses and three washes of 20 min in PBST. Ovaries were mounted on a glass slide in Prolong Gold antifade reagent (Life Technologies), and ovarioles were separated fully during mounting.

Single-Molecule FISH

Single-molecule FISH was performed using Stellaris (Biosearch Technologies) oligonucleotide probes 20 nt in length complementary to the *grk* transcript (CG17610, 48 probes), conjugated to CAL Fluor Red 590. Fixed ovaries were washed for 10 min in 50% PBST, 50% Hybe− solution (10% deionized formamide, 2× SSC, 2 mM ribonucleoside vanadyl complex, and 0.02 BSA) and then 10 min in Hybe− solution before pre-hybridizing for 1 hr in Hybe+ solution (10% deionized formamide, 2× SSC, 2 mM ribonucleoside vanadyl complex, 0.02% BSA, and 10% dextran sulfate). Hybridization of probes was performed for 16–24 hr at a concentration of 25 nM in Hybe+ solution at 37°C. Ovaries were washed twice for 1 hr in wash buffer (15% deionized formamide and 2× SSC) and mounted in Prolong Gold for imaging.

SUPPLEMENTAL INFORMATION

Supplemental Information includes five figures and can be found with this article online at http://dx.doi.org/10.1016/j.celrep.2016.02.038.

AUTHOR CONTRIBUTIONS

A.D., R.M.P., C.R., T.T.W., and I.D. provided the intellectual basis of the work, designed the experiments, and wrote and edited the manuscript. A.D., R.M.P, and T.T.W. performed the experiments. R.M.P. and C.R. provided technical expertise for imaging and data analysis.

ACKNOWLEDGMENTS

We are grateful to Bram Herpers (Leiden) for the egg chamber IEM sections; A. Ephrussi (EMBL), A. Nakamura (RIKEN, Japan), P. Macdonald (University of Texas at Austin), M. Buszczak (University of Texas SouthWestern Medical Center), T. Schüpbach (Princeton University) and J. van Minne (University of Calgary) for fly stocks, antibodies, and reagents; the Bloomington Stock Center (Indiana University) for fly stocks; FlyBase for database information; P. Schedl (Princeton University) and T. Schupbach (Princeton University) for experimental advice; L. Schermelleh (Department of Biochemistry, University of Oxford) and Micron Oxford for help and advice with 3D-SIM; James Halstead (Friedrich Miescher Institute for Biomedical Research), Tatjana Trcek (NYC School of Medicine), and Lu Yang (Department of Biochemistry, University of Oxford) for assistance in optimization of protocols; and David Ish-Horowicz for critical reading of the manuscript. This work was supported by a studentship from the Wellcome Trust (grant 097304 to A.D.), a Wellcome Trust Senior Research Fellowship (grant 096144 to I.D and supporting R.M.P), the University of Cambridge, ISSF (grant 097814 to T.T.W), and Wellcome Trust Strategic Awards 091911 and 107457 supporting advanced microscopy at Micron Oxford (http://micronoxford.com).

Received: September 8, 2015
Revised: December 18, 2015
Accepted: February 3, 2016
Published: March 3, 2016

REFERENCES

Bertrand, E., Chartrand, P., Schaefer, M., Shenoy, S.M., Singer, R.H., and Long, R.M. (1998). Localization of ASH1 mRNA particles in living yeast. Mol. Cell *2*, 437–445.

Besse, F., and Ephrussi, A. (2008). Translational control of localized mRNAs: restricting protein synthesis in space and time. Nat. Rev. Mol. Cell Biol. *9*, 971–980.

Besse, F., López de Quinto, S., Marchand, V., Trucco, A., and Ephrussi, A. (2009). Drosophila PTB promotes formation of high-order RNP particles and represses oskar translation. Genes Dev. *23*, 195–207.

Bökel, C., Dass, S., Wilsch-Bräuninger, M., and Roth, S. (2006). Drosophila Cornichon acts as cargo receptor for ER export of the TGFalpha-like growth factor Gurken. Development *133*, 459–470.

Buszczak, M., Paterno, S., Lighthouse, D., Bachman, J., Planck, J., Owen, S., Skora, A.D., Nystul, T.G., Ohlstein, B., Allen, A., et al. (2007). The carnegie protein trap library: a versatile tool for Drosophila developmental studies. Genetics *175*, 1505–1531.

Cáceres, L., and Nilson, L.A. (2009). Translational repression of gurken mRNA in the Drosophila oocyte requires the hnRNP Squid in the nurse cells. Dev. Biol. *326*, 327–334.

Cao, Q., and Richter, J.D. (2002). Dissolution of the maskin-eIF4E complex by cytoplasmic polyadenylation and poly(A)-binding protein controls cyclin B1 mRNA translation and oocyte maturation. EMBO J. *21*, 3852–3862.

Castagnetti, S., and Ephrussi, A. (2003). Orb and a long poly(A) tail are required for efficient oskar translation at the posterior pole of the Drosophila oocyte. Development *130*, 835–843.

Chang, J.S., Tan, L., and Schedl, P. (1999). The Drosophila CPEB homolog, orb, is required for oskar protein expression in oocytes. Dev. Biol. *215*, 91–106.

Chang, J.S., Tan, L., Wolf, M.R., and Schedl, P. (2001). Functioning of the Drosophila orb gene in gurken mRNA localization and translation. Development *128*, 3169–3177.

Chekulaeva, M., Hentze, M.W., and Ephrussi, A. (2006). Bruno acts as a dual repressor of oskar translation, promoting mRNA oligomerization and formation of silencing particles. Cell *124*, 521–533.

Christerson, L.B., and McKearin, D.M. (1994). orb is required for anteroposterior and dorsoventral patterning during Drosophila oogenesis. Genes Dev. *8*, 614–628.

Clouse, K.N., Ferguson, S.B., and Schüpbach, T. (2008). Squid, Cup, and PABP55B function together to regulate gurken translation in Drosophila. Dev. Biol. *313*, 713–724.

Costa, A., Wang, Y., Dockendorff, T.C., Erdjument-Bromage, H., Tempst, P., Schedl, P., and Jongens, T.A. (2005). The Drosophila fragile X protein functions as a negative regulator in the orb autoregulatory pathway. Dev. Cell *8*, 331–342.

Crucs, S., Chatterjee, S., and Gavis, E.R. (2000). Overlapping but distinct RNA elements control repression and activation of nanos translation. Mol. Cell *5*, 457–467.

Decker, C.J., and Parker, R. (2012). P-bodies and stress granules: possible roles in the control of translation and mRNA degradation. Cold Spring Harb. Perspect. Biol. *4*, a012286.

Delanoue, R., Herpers, B., Soetaert, J., Davis, I., and Rabouille, C. (2007). Drosophila Squid/hnRNP helps Dynein switch from a gurken mRNA transport motor to an ultrastructural static anchor in sponge bodies. Dev. Cell *13*, 523–538.

Dobbie, I.M., King, E., Parton, R.M., Carlton, P.M., Sedat, J.W., Swedlow, J.R., and Davis, I. (2011). OMX: a new platform for multimodal, multichannel widefield imaging. Cold Spring Harb. Protoc. *2011*, 899–909.

Ephrussi, A., Dickinson, L.K., and Lehmann, R. (1991). Oskar organizes the germ plasm and directs localization of the posterior determinant nanos. Cell *66*, 37–50.

Forrest, K.M., and Gavis, E.R. (2003). Live imaging of endogenous RNA reveals a diffusion and entrapment mechanism for nanos mRNA localization in Drosophila. Curr. Biol. *13*, 1159–1168.

Fox, C.A., Sheets, M.D., and Wickens, M.P. (1989). Poly(A) addition during maturation of frog oocytes: distinct nuclear and cytoplasmic activities and regulation by the sequence UUUUUAU. Genes Dev. *3* (*12B*), 2151–2162.

Gamberi, C., Peterson, D.S., He, L., and Gottlieb, E. (2002). An anterior function for the Drosophila posterior determinant Pumilio. Development *129*, 2699–2710.

Hachet, O., and Ephrussi, A. (2004). Splicing of oskar RNA in the nucleus is coupled to its cytoplasmic localization. Nature *428*, 959–963.

Hake, L.E., and Richter, J.D. (1994). CPEB is a specificity factor that mediates cytoplasmic polyadenylation during Xenopus oocyte maturation. Cell *79*, 617–627.

Hawkins, N.C., Thorpe, J., and Schüpbach, T. (1996). Encore, a gene required for the regulation of germ line mitosis and oocyte differentiation during Drosophila oogenesis. Development *122*, 281–290.

Hawkins, N.C., Van Buskirk, C., Grossniklaus, U., and Schüpbach, T. (1997). Post-transcriptional regulation of gurken by encore is required for axis determination in Drosophila. Development *124*, 4801–4810.

Herpers, B., Xanthakis, D., and Rabouille, C. (2010). ISH-IEM: a sensitive method to detect endogenous mRNAs at the ultrastructural level. Nat. Protoc. *5*, 678–687.

Igreja, C., and Izaurralde, E. (2011). CUP promotes deadenylation and inhibits decapping of mRNA targets. Genes Dev. *25*, 1955–1967.

Ivshina, M., Lasko, P., and Richter, J.D. (2014). Cytoplasmic polyadenylation element binding proteins in development, health, and disease. Annu. Rev. Cell Dev. Biol. *30*, 393–415.

Jackson, R.J., Hellen, C.U.T., and Pestova, T.V. (2010). The mechanism of eukaryotic translation initiation and principles of its regulation. Nat. Rev. Mol. Cell Biol. *11*, 113–127.

Jaramillo, A.M., Weil, T.T., Goodhouse, J., Gavis, E.R., and Schupbach, T. (2008). The dynamics of fluorescently labeled endogenous gurken mRNA in Drosophila. J. Cell Sci. *121*, 887–894.

Juge, F., Zaessinger, S., Temme, C., Wahle, E., and Simonelig, M. (2002). Control of poly(A) polymerase level is essential to cytoplasmic polyadenylation and early development in Drosophila. EMBO J. *21*, 6603–6613.

Kalifa, Y., Huang, T., Rosen, L.N., Chatterjee, S., and Gavis, E.R. (2006). Glorund, a Drosophila hnRNP F/H homolog, is an ovarian repressor of nanos translation. Dev. Cell *10*, 291–301.

Kamenska, A., Lu, W.-T., Kubacka, D., Broomhead, H., Minshall, N., Bushell, M., and Standart, N. (2014). Human 4E-T represses translation of bound mRNAs and enhances microRNA-mediated silencing. Nucleic Acids Res. *42*, 3298–3313.

Karpen, G.H., and Spradling, A.C. (1992). Analysis of subtelomeric heterochromatin in the Drosophila minichromosome Dp1187 by single P element insertional mutagenesis. Genetics *132*, 737–753.

Kelley, R.L. (1993). Initial organization of the Drosophila dorsoventral axis depends on an RNA-binding protein encoded by the squid gene. Genes Dev. *7*, 948–960.

Kim, J.H., and Richter, J.D. (2006). Opposing polymerase-deadenylase activities regulate cytoplasmic polyadenylation. Mol. Cell *24*, 173–183.

Kim-Ha, J., Kerr, K., and Macdonald, P.M. (1995). Translational regulation of oskar mRNA by bruno, an ovarian RNA-binding protein, is essential. Cell *81*, 403–412.

Lantz, V., Ambrosio, L., and Schedl, P. (1992). The Drosophila orb gene is predicted to encode sex-specific germline RNA-binding proteins and has localized transcripts in ovaries and early embryos. Development *115*, 75–88.

Li, W., Klovstad, M., and Schüpbach, T. (2014). Repression of Gurken translation by a meiotic checkpoint in Drosophila oogenesis is suppressed by a reduction in the dose of eIF1A. Development *141*, 3910–3921.

Little, S.C., Sinsimer, K.S., Lee, J.J., Wieschaus, E.F., and Gavis, E.R. (2015). Independent and coordinate trafficking of single Drosophila germ plasm mRNAs. Nat. Cell Biol. *17*, 558–568.

Mansfield, J.H., Wilhelm, J.E., and Hazelrigg, T. (2002). Ypsilon Schachtel, a Drosophila Y-box protein, acts antagonistically to Orb in the oskar mRNA localization and translation pathway. Development *129*, 197–209.

McDermott, S.M., Meignin, C., Rappsilber, J., and Davis, I. (2012). Drosophila Syncrip binds the gurken mRNA localisation signal and regulates localised transcripts during axis specification. Biol. Open *1*, 488–497.

Medioni, C., Mowry, K., and Besse, F. (2012). Principles and roles of mRNA localization in animal development. Development *139*, 3263–3276.

Minshall, N., Reiter, M.H., Weil, D., and Standart, N. (2007). CPEB interacts with an ovary-specific eIF4E and 4E-T in early Xenopus oocytes. J. Biol. Chem. *282*, 37389–37401.

Nakamura, A., Amikura, R., Hanyu, K., and Kobayashi, S. (2001). Me31B silences translation of oocyte-localizing RNAs through the formation of cytoplasmic RNP complex during Drosophila oogenesis. Development *128*, 3233–3242.

Nakamura, A., Sato, K., and Hanyu-Nakamura, K. (2004). Drosophila cup is an eIF4E binding protein that associates with Bruno and regulates oskar mRNA translation in oogenesis. Dev. Cell *6*, 69–78.

Nelson, M.R., Leidal, A.M., and Smibert, C.A. (2004). Drosophila Cup is an eIF4E-binding protein that functions in Smaug-mediated translational repression. EMBO J. *23*, 150–159.

Neuman-Silberberg, F.S., and Schüpbach, T. (1993). The Drosophila dorsoventral patterning gene gurken produces a dorsally localized RNA and encodes a TGF alpha-like protein. Cell *75*, 165–174.

Neuman-Silberberg, F.S., and Schüpbach, T. (1994). Dorsoventral axis formation in Drosophila depends on the correct dosage of the gene gurken. Development *120*, 2457–2463.

Norvell, A., Kelley, R.L., Wehr, K., and Schüpbach, T. (1999). Specific isoforms of squid, a Drosophila hnRNP, perform distinct roles in Gurken localization during oogenesis. Genes Dev. *13*, 864–876.

Norvell, A., Wong, J., Randolph, K., and Thompson, L. (2015). Wispy and Orb cooperate in the cytoplasmic polyadenylation of localized gurken mRNA. Dev. Dyn. *244*, 1276–1285.

Parton, R.M., Hamilton, R.S., Ball, G., Yang, L., Cullen, C.F., Lu, W., Ohkura, H., and Davis, I. (2011). A PAR-1-dependent orientation gradient of dynamic microtubules directs posterior cargo transport in the Drosophila oocyte. J. Cell Biol. *194*, 121–135.

Queenan, A.M., Barcelo, G., Van Buskirk, C., and Schüpbach, T. (1999). The transmembrane region of Gurken is not required for biological activity, but is necessary for transport to the oocyte membrane in Drosophila. Mech. Dev. *89*, 35–42.

Reveal, B., Yan, N., Snee, M.J., Pai, C.I., Gim, Y., and Macdonald, P.M. (2010). BREs mediate both repression and activation of oskar mRNA translation and act in trans. Dev. Cell *18*, 496–502.

Reveal, B., Garcia, C., Ellington, A., and Macdonald, P.M. (2011). Multiple RNA binding domains of Bruno confer recognition of diverse binding sites for translational repression. RNA Biol. *8*, 1047–1060.

Richter, J.D., and Lasko, P. (2011). Translational control in oocyte development. Cold Spring Harb. Perspect. Biol. *3*, a002758.

Richter, J.D., and Sonenberg, N. (2005). Regulation of cap-dependent translation by eIF4E inhibitory proteins. Nature *433*, 477–480.

Sarkissian, M., Méndez, R., and Richter, J.D. (2004). Progesterone and insulin stimulation of CPEB-dependent polyadenylation is regulated by Aurora A and glycogen synthase kinase-3. Genes Dev. *18*, 48–61.

Schermelleh, L., Carlton, P.M., Haase, S., Shao, L., Winoto, L., Kner, P., Burke, B., Cardoso, M.C., Agard, D.A., Gustafsson, M.G.L., et al. (2008). Subdiffraction multicolor imaging of the nuclear periphery with 3D structured illumination microscopy. Science *320*, 1332–1336.

Schüpbach, T., and Wieschaus, E. (1991). Female sterile mutations on the second chromosome of Drosophila melanogaster. II. Mutations blocking oogenesis or altering egg morphology. Genetics *129*, 1119–1136.

Snee, M.J., and Macdonald, P.M. (2004). Live imaging of nuage and polar granules: evidence against a precursor-product relationship and a novel role for Oskar in stabilization of polar granule components. J. Cell Sci. *117*, 2109–2120.

Stebbins-Boaz, B., Cao, Q., de Moor, C.H., Mendez, R., and Richter, J.D. (1999). Maskin is a CPEB-associated factor that transiently interacts with eIF-4E. Mol. Cell *4*, 1017–1027.

Tan, L., Chang, J.S., Costa, A., and Schedl, P. (2001). An autoregulatory feedback loop directs the localized expression of the Drosophila CPEB protein Orb in the developing oocyte. Development *128*, 1159–1169.

Van Buskirk, C., Hawkins, N.C., and Schüpbach, T. (2000). Encore is a member of a novel family of proteins and affects multiple processes in Drosophila oogenesis. Development *127*, 4753–4762.

Weil, T.T. (2014). mRNA localization in the Drosophila germline. RNA Biol. *11*, 1010–1018.

Weil, T.T., Parton, R.M., Herpers, B., Soetaert, J., Veenendaal, T., Xanthakis, D., Dobbie, I.M., Halstead, J.M., Hayashi, R., Rabouille, C., and Davis, I. (2012). Drosophila patterning is established by differential association of mRNAs with P bodies. Nat. Cell Biol. *14*, 1305–1313.

Wilhelm, J.E., Hilton, M., Amos, Q., and Henzel, W.J. (2003). Cup is an eIF4E binding protein required for both the translational repression of oskar and the recruitment of Barentsz. J. Cell Biol. *163*, 1197–1204.

Wong, L.C., and Schedl, P. (2011). Cup blocks the precocious activation of the orb autoregulatory loop. PLoS ONE *6*, e28261.

Wong, L.C., Costa, A., McLeod, I., Sarkeshik, A., Yates, J., 3rd, Kyin, S., Perlman, D., and Schedl, P. (2011). The functioning of the Drosophila CPEB protein Orb is regulated by phosphorylation and requires casein kinase 2 activity. PLoS ONE *6*, e24355.

Yan, N., and Macdonald, P.M. (2004). Genetic interactions of Drosophila melanogaster arrest reveal roles for translational repressor Bruno in accumulation of Gurken and activity of Delta. Genetics *168*, 1433–1442.

Zaessinger, S., Busseau, I., and Simonelig, M. (2006). Oskar allows nanos mRNA translation in Drosophila embryos by preventing its deadenylation by Smaug/CCR4. Development *133*, 4573–4583.

Cell Reports, Volume *14*

Supplemental Information

Localized Translation of *gurken*/*TGF-*α mRNA during Axis Specification Is Controlled by Access to Orb/CPEB on Processing Bodies

Alexander Davidson, Richard M. Parton, Catherine Rabouille, Timothy T. Weil, and Ilan Davis

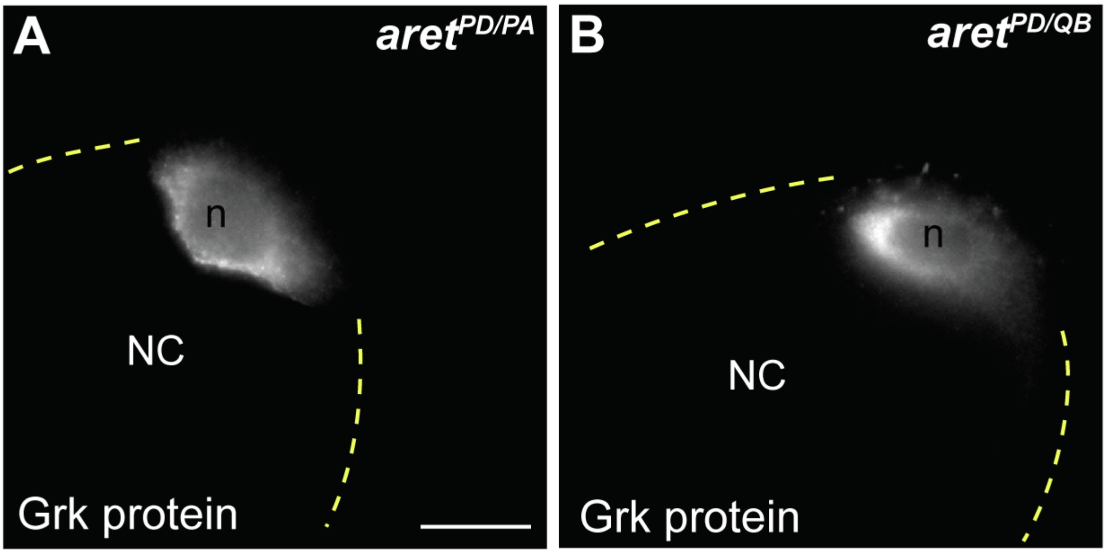

Figure S1. Grk protein is not expressed in the nurse cells in a number of *aret* allelic combinations, related to Figure 1

(A-B) In *aret* mutants, *WT* Grk expression is observed, with no ectopic staining in the nurse cells (A, n = 60; B, n = 60). Scale bar 15 µm; NC, Nurse cells; n, oocyte nucleus. Dashed yellow lines indicate the edges of the egg chamber.

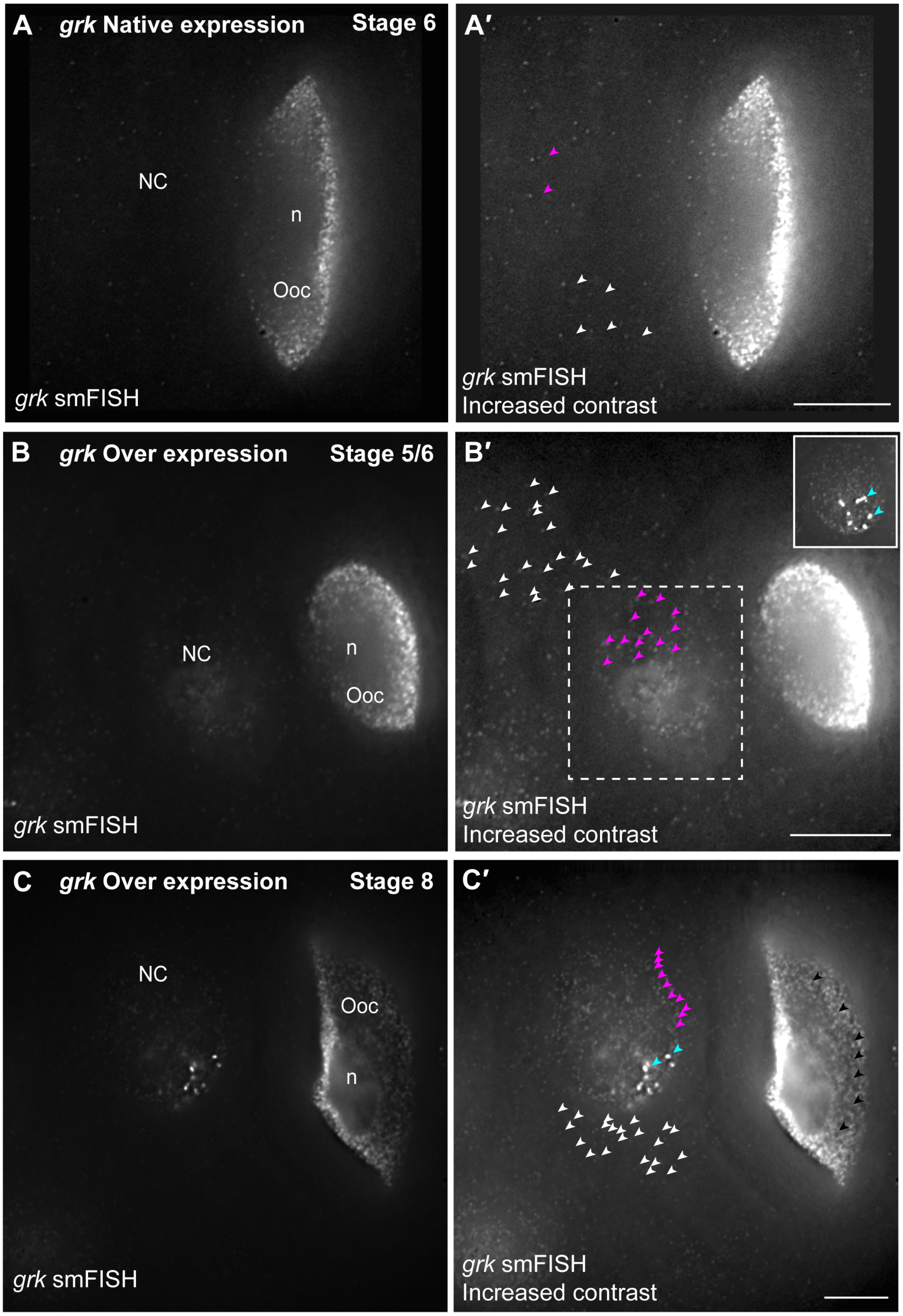

Figure S2. High levels of full-length *grk* transcripts are produced in nurse cells and egg chambers using the UAS-Gal4 system, related to Figure 1

(A-A′) *grk* sm-FISH on *WT* egg chambers showing low numbers of transcripts in NC compared to the oocyte, (A′) is over contrasted to show the weak signal in NC. Arrowheads identify different populations of transcripts in the NC cytoplasm (white) and NC nucleus (magenta). (B-C′)

Overexpression of *grk* driven by the UAS-Gal4 system in nurse cells and oocytes at both early (B-B′) and mid oogenesis (C-C′). Inset in (B′) a different focal plane, corresponding to the dashed box in the main panel, highlighting abundant nascent transcripts in the NC nucleus with over-expression. The increase in numbers of transcript foci between *WT* and driven UASp-*grk* was quantified for regions of interest in the cytoplasm of NC from stage 8 egg chambers: *WT* mean count = 11.28 $\pm$1.3 SEM; driven UASp-*grk* mean count = 29.14 $\pm$3.3 SEM, n = 14, P = 0.00009 (Student's t-test). Arrowheads identify different populations of transcripts in the NC cytoplasm (white), NC nucleus (magenta), nascent transcripts (cyan) and transcripts in the oocyte (black). Scale bars 15 µm; NC, Nurse cells; Ooc, Oocyte; n, oocyte nucleus.

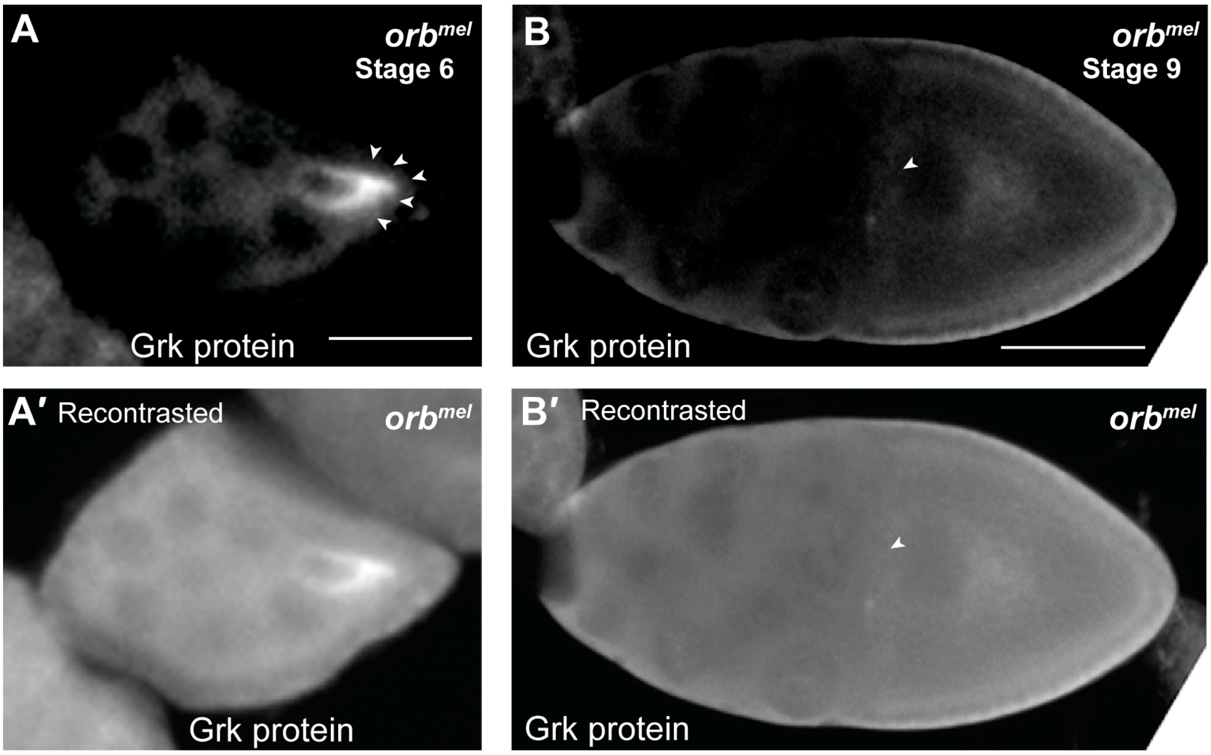

Figure S3. Grk protein is not expressed in late stage *orb^{mel}* egg chambers, related to Figure 1

(A-B′) Grk antibody staining in orb^{mel} egg chambers. (A-A′) In stage 6 orb^{mel} egg chambers, Grk protein is expressed in the oocyte (n = 5). (B-B′) In stage 9 orb^{mel} egg chambers, Grk protein is not expressed in the oocyte, arrowhead in (B) indicates the DA where expression would be expected (n = 5). (A′ and B′) Over-contrasted images highlighting the lack of Grk. Scale bars 25 μm (A), 50 μm (B).

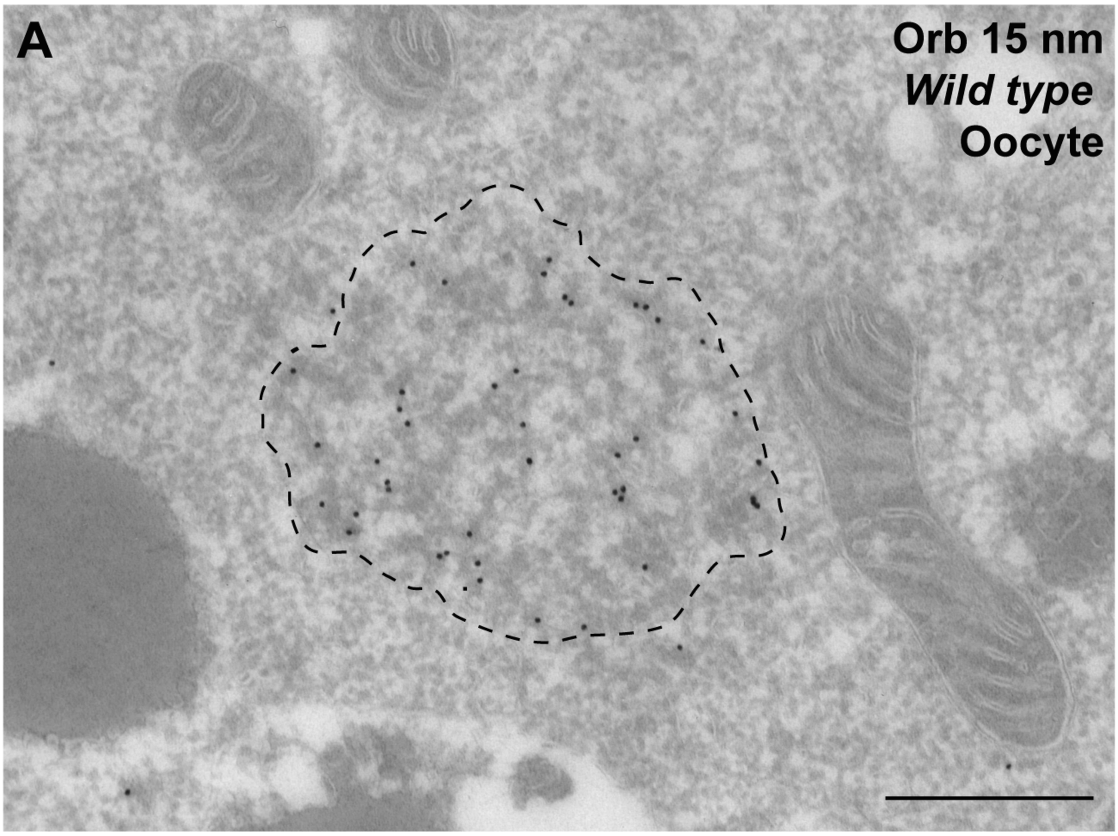

Figure S4. P bodies throughout the oocyte contain Orb protein, related to Figure 3

(A) Orb protein detection by IEM on ultra-thin frozen sections of nurse cells. Anti-Orb (15nm gold) is present in P bodies throughout the oocyte. Dashed black lines mark the edge of the P body. Scale bar: 500nm.

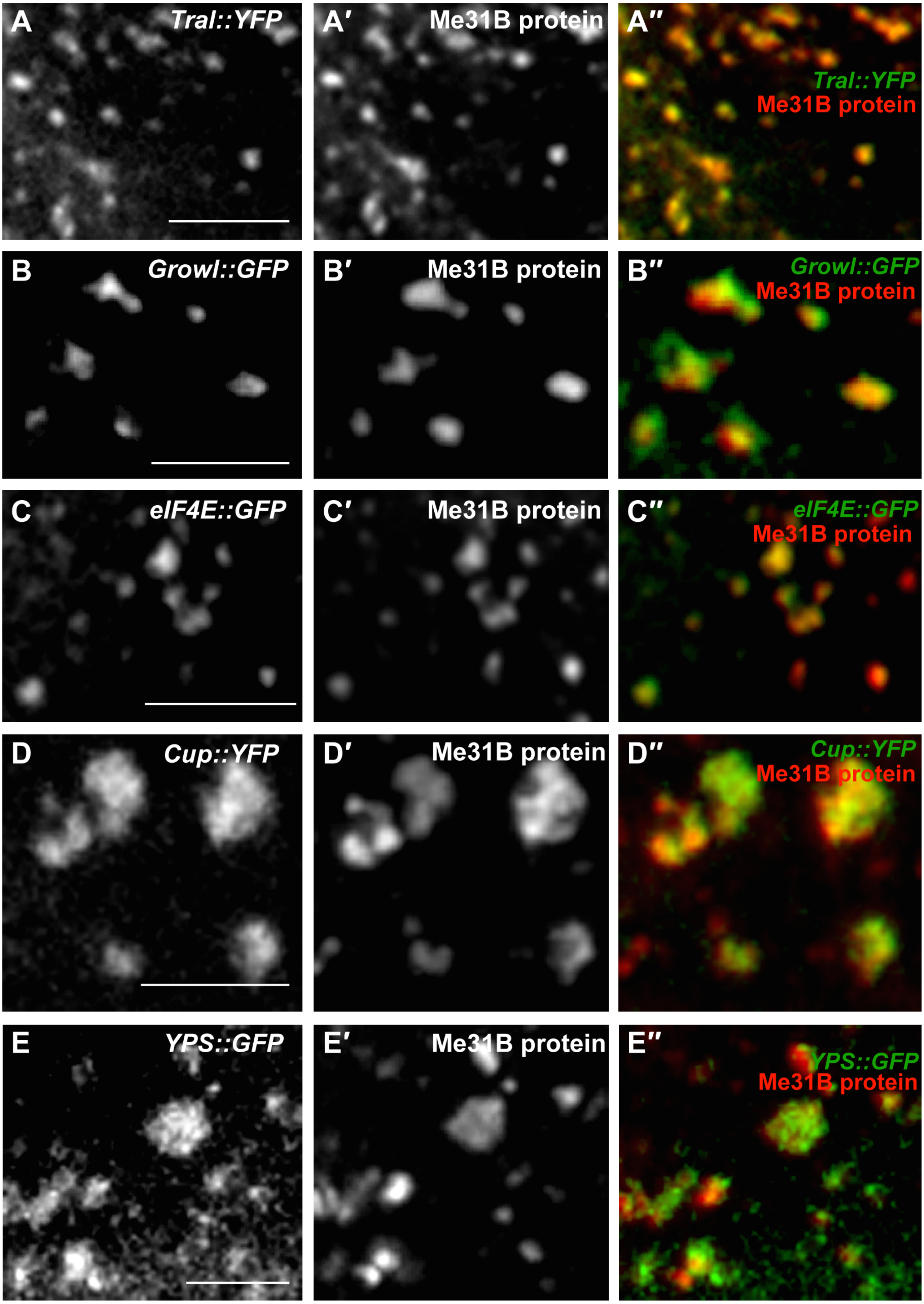
A
Tral::YFP
A′
Me31B protein
A″
Tral::YFP
Me31B protein
B
Growl::GFP
B′
Me31B protein
B″
Growl::GFP
Me31B protein
C
eIF4E::GFP
C′
Me31B protein
C″
eIF4E::GFP
Me31B protein
D
Cup::YFP
D′
Me31B protein
D″
Cup::YFP
Me31B protein
E
YPS::GFP
E′
Me31B protein
E″
YPS::GFP
Me31B protein

Figure S5. Nurse cell P bodies visualised by immunofluorescence have a similar protein complement to those in the oocyte, related to Figure 3

(A-E″) Protein detection by immunofluorescence on whole egg chambers. Tral (A), Growl (B), eIF4E (C), Cup (D), and YPS (E), labelled with fluorescent protein traps, colocalize extensively with Me31B antibody staining (A″,B″,D″,E″) in the nurse cell cytoplasm (n = 60). Note: some bodies are brighter in one channel, making a direct comparison difficult. All images were deconvolved and are 2 μm maximum intensity projections. Scale bars: 5 μm (A, C), 2.5 μm (B, D, E).
